# Synthesis and Characterization of Thiophene‐based Donor–Acceptor–Donor Heptameric Ligands for Spectral Assignment of Polymorphic Amyloid‐β Deposits

**DOI:** 10.1002/chem.201905612

**Published:** 2020-05-15

**Authors:** Linda Lantz, Hamid Shirani, Therése Klingstedt, K. Peter R. Nilsson

**Affiliations:** ^1^ Division of Chemistry Department of Physics, Chemistry and Biology Linköping University 581 83 Linköping Sweden

**Keywords:** Alzheimer's disease, fluorescence, luminescent conjugated oligothiophenes, protein aggregates, β-amyloid

## Abstract

Protein deposits are associated with many devastating diseases and fluorescent ligands able to visualize these pathological entities are essential. Here, we report the synthesis of thiophene‐based donor–acceptor–donor heptameric ligands that can be utilized for spectral assignment of distinct amyloid‐β (Aβ) aggregates, one of the pathological hallmarks in Alzheimer's disease. The ability of the ligands to selectively distinguish Aβ deposits was abolished when the chemical composition of the ligands was altered. Our findings provide the structural and functional basis for the development of new fluorescent ligands that can distinguish between aggregated proteinaceous species consisting of the same peptide or protein. In addition, such ligands might aid in interpreting the potential role of polymorphic Aβ deposits in the pathogenesis of Alzheimer's disease.

## Graphical abstract

**Head or tail**: Distinct periodicity of anionic charges along the conjugated backbone is an important chemical determinant for achieving thiophene‐based donor–acceptor–donor heptameric ligands for spectral assignment of different amyloid‐β deposits. Having the acetate side chains of the central trimeric thiophene‐BTD‐thiophene unit tail‐to‐tail instead of head‐to‐head greatly reduced the ability of the ligand to distinguish Aβ morphotypes.

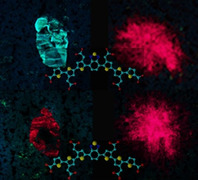

## Introduction

Aggregated proteins are observed as pathological hallmarks in many devastating diseases, such as Alzheimer's disease (AD), and ligands that selectively detect these protein deposits are of great interest.^1^, ^2^, ^3^ A variety of ligands targeting the common repetitive cross β‐pleated sheet fibrillar structure of these proteinaceous species have been reported and the strategy of utilizing small chemically defined fluorescent molecules has successfully been employed to identify amyloid‐β (Aβ) deposits, one of the major pathological hallmarks in AD.^4^, ^5^, ^6^, ^7^, ^8^, ^9^, ^10^ However, most conventional ligands detect disease‐associated protein aggregates in general and several studies have revealed a striking conformational polymorphism in many disease‐associated protein aggregates comprised of the same peptide or protein.^11^, ^12^, ^13^, ^14^, ^15^, ^16^, ^17^, ^18^ Hence, the next scientific challenge is to develop ligands that can differentiate such polymorphic protein aggregates.

The prion protein is a classic example of how an identical primary sequence of amino acids can misfold into distinct aggregate morphotypes.^11^, ^12^, ^13^, ^14^ Lately, an inter‐subject variability of Aβ deposits has also been found in familial and sporadic AD.^19^, ^20^, ^21^ Likewise, distinct age dependent Aβ deposits were observed in transgenic mouse models, suggesting that different aggregate species of Aβ are present during different stages of the pathological process.^22^, ^23^ In addition, distinct conformations of Aβ aggregates have also been described as formed either spontaneously from recombinant Aβ or after seeding of recombinant Aβ by aggregates from AD brains.^24^, ^25^, ^26^, ^27^, ^28^ Notably, seeding with Aβ aggregates extracted from two AD patients with distinct clinical history and pathology resulted in fibrils with two different structures, implying a correlation between aggregate structure and disease progression.^28^ Thus, ligands that can differentiate different types of Aβ aggregates are essential as such molecular agents will aid in accurate clinical diagnostics of AD, as well as assist in deciphering the impact of polymorphic Aβ deposits in the pathogenesis of AD.

**Figure 1:**
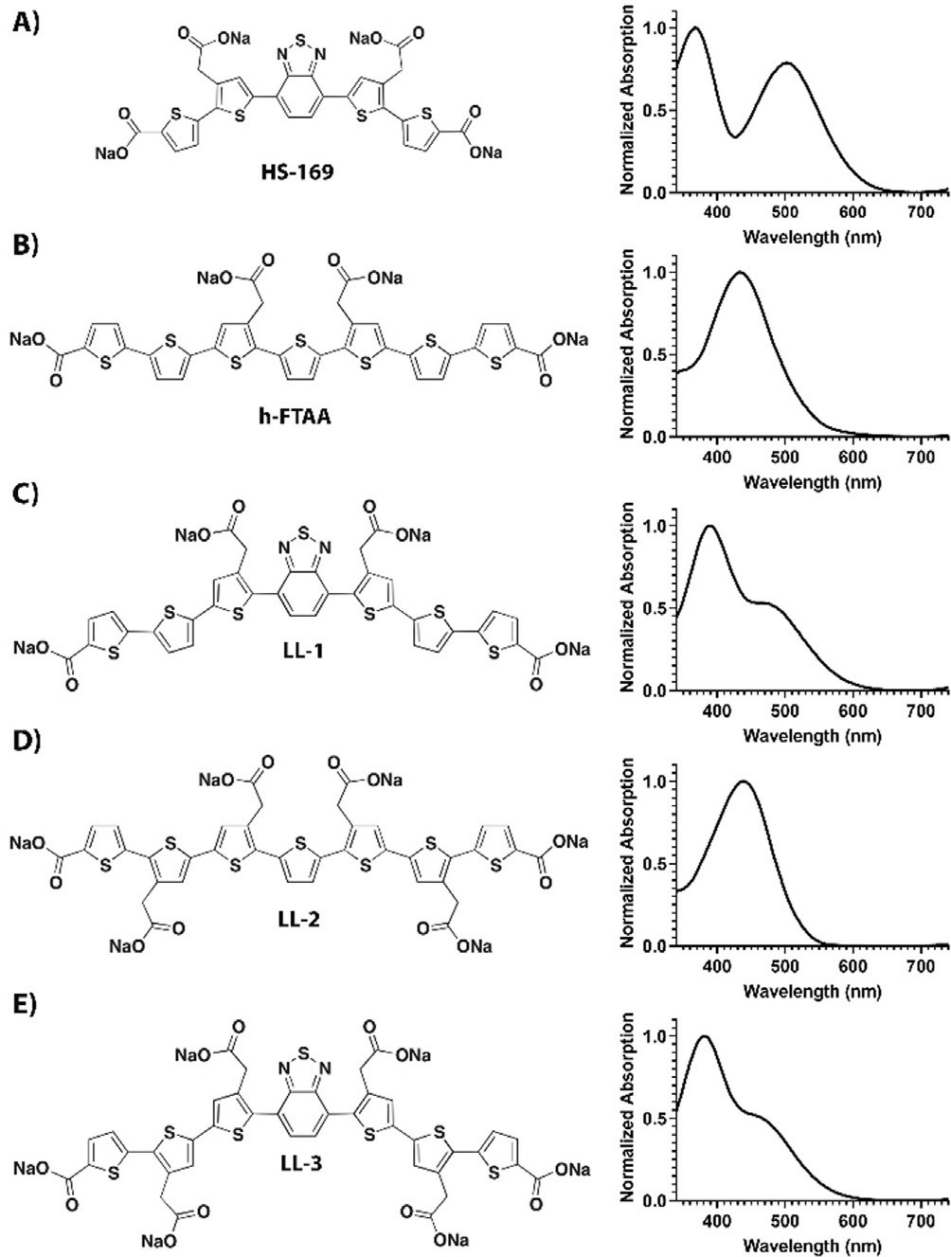
Chemical structure and absorption spectra (30 μm ligand in PBS pH 7.4) of the thiophene‐based pentameric and heptameric ligands. HS‐169 (A), h‐FTAA (B), LL‐1 (C), LL‐2 (D) and LL‐3 (E).

Luminescent conjugated oligothiophenes (LCOs) are thiophene‐based ligands that have been established as a class of ligands for superior recognition and spectral assignment of disease‐associated protein aggregates, including different polymorphic Aβ aggregates.^19^, ^22^, ^23^, ^29^, ^30^, ^31^, ^32^, ^33^ Owing to their electronically delocalized conjugated thiophene backbones, LCOs exhibit intrinsic conformational dependent fluorescence characteristics that can be recorded by different modes of detection.^33^ Lately, a thiophene‐based pentameric ligand, HS‐169 (Figure 1 A), with donor–acceptor–donor (D–A–D) type electronic structure was also presented.^34^ The D–A–D photophysical characteristics were obtained by replacing the central thiophene moiety with 2,1,3‐benzothiadiazole (BTD), rendering a pentameric ligand where electron rich bithiophene units act as donors and the electron‐withdrawing BTD as acceptor. HS‐169 selectively identified Aβ pathology in human AD brain tissue sections and the ligand displayed similar near‐infra red emission bound to Aβ core plaques or cerebral β‐amyloid angiopathy (CAA).^34^ Furthermore, HS‐169 enabled optical assignment of specific carbohydrates, cellulose or starch, in plant tissue and this spectral distinction could not be obtained by the corresponding oligothiophene derivative.^35^ Thus, the D–A–D electronic structure of HS‐169 was crucial for distinguishing specific carbohydrates.

**Scheme 1:**
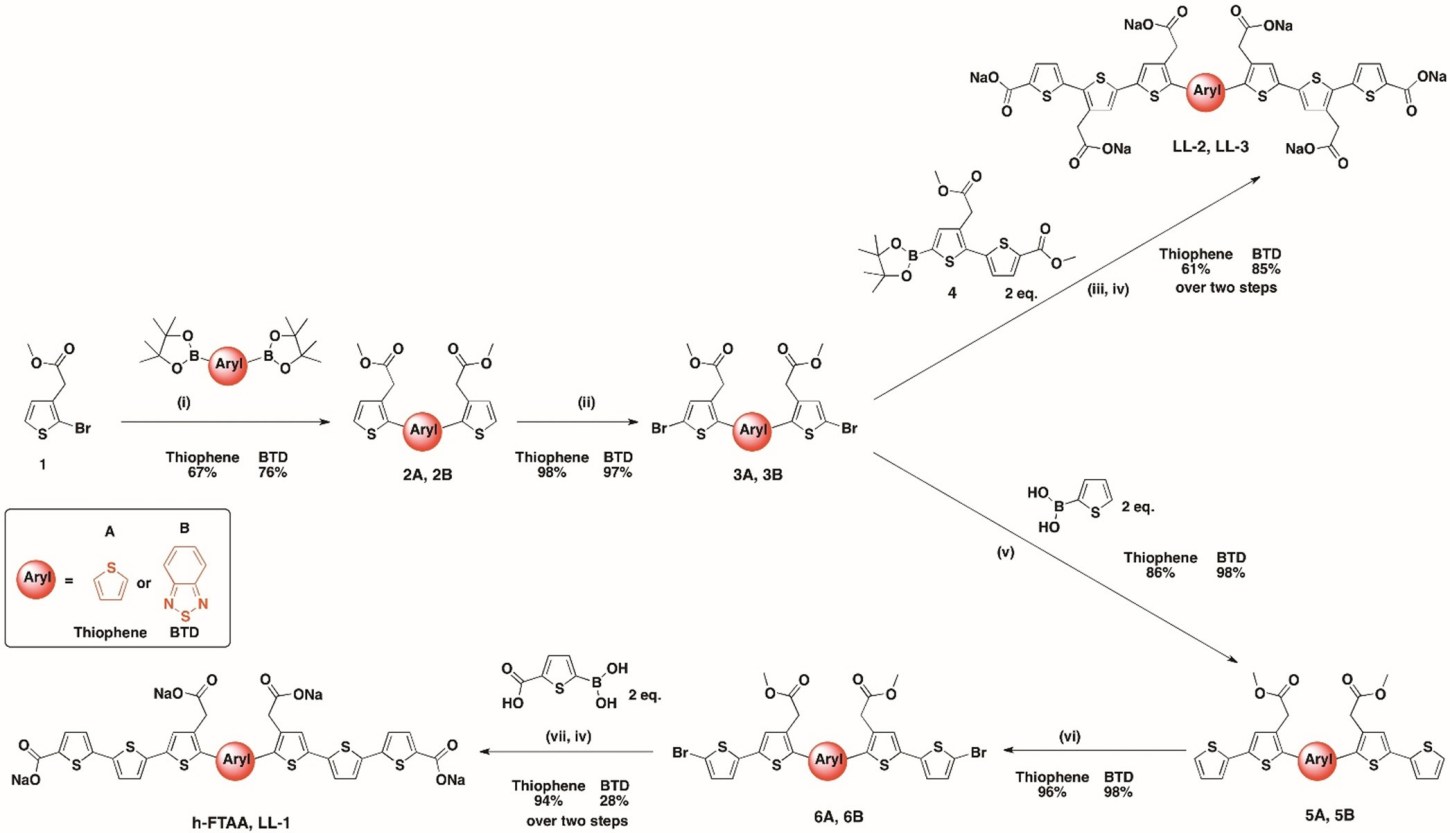
Synthesis of h‐FTAA, LL‐1, LL‐2 and LL‐3: Reagents and conditions: (i) PEPPSI™‐IPr, K_2_CO_3_, 1,4‐dioxane/toluene/MeOH (1:1:1), 80 °C, 2 h; (ii) NBS, DMF/chloroform (1:1), −15 °C to r.t., 18 h; (iii) PEPPSI™‐IPr, K_2_CO_3_, 1,4‐dioxane, 80 °C, 2 h; (iv) NaOH (1 m, aq.), 1,4‐dioxane, 40 °C; (v) PEPPSI™‐IPr, K_2_CO_3_, toluene/MeOH (1:1), MW 100 °C, 20 min; (vi) NBS, chloroform, −15 °C to r.t., 18 h; (vii) PEPPSI™‐IPr, K_2_CO_3_, 1,4‐dioxane/toluene/MeOH (1:1:1), MW 100 °C, 40 min.

Herein, we present the synthesis and characterization of anionic heptameric oligothiophene derivatives having thiophene or BTD as the central heterocyclic moiety (Figure 1, Scheme 1). The ligands were utilized for spectral assignment of recombinant Aβ fibrils and Aβ deposits in brain sections from transgenic mice with AD‐like pathology. The ligands selectively identified Aβ aggregates and subtle changes in the chemical composition of the ligands were shown to eliminate their capacity for spectral separation of specific Aβ deposits. Thus, these findings might aid in the chemical design of ligands recognizing different aggregated proteinaceous species consisting of a distinct protein.

## Results and Discussion

### Synthesis and optical characterization of the ligands

To achieve a variety of anionic heptameric oligothiophene derivatives having thiophene or BTD as the central heterocyclic moiety, we started with a previously reported monobrominated thiophene building block^30^ and boronic acid pinacol esters of thiophene and BTD (Scheme 1). By applying palladium‐mediated Suzuki–Miyaura cross‐coupling reactions and bromination with *N*‐bromosuccinimide (NBS) in DMF/chloroform, two distinct dibrominated trimer building blocks were achieved in affordable yields. From these trimeric building blocks, four different heptameric ligands, h‐FTAA, LL‐1, LL‐2 and LL‐3, were generated by applying sequential symmetric addition of different thiophene or bithiophene units through Suzuki–Miyaura cross‐coupling reactions and regioselective brominations followed by removal of protecting groups to have anionic side chain functionalities (Scheme 1 and Figure 1). LL‐1 (Figure 1 C) resembles the previously synthesized heptameric oligothiophene, h‐FTAA (Figure 1 B), with a seven units long backbone and four carboxylate side chain functionalities at distinct positions, as well as a central BTD moiety instead of thiophene. Similar to h‐FTAA, thiophenes constitute the entire backbone of LL‐2 (Figure 1 D), though, this ligand comprises six carboxylate side chain functionalities which render a higher negative net charge compared to both h‐FTAA and LL‐1. The ligand denoted LL‐3 (Figure 1 E) is the corresponding BTD analogue to LL‐2 and contains six anionic substituents, as well as a central BTD moiety. In addition to the heptameric ligands described above, the previously reported pentameric thiophene‐based ligand with a central BTD moiety, HS‐169 (Figure 1 A),^34^ was included in the study.

When diluted in phosphate buffered saline (PBS, 20 mm Na‐phosphate, 150 mm NaCl, pH 7.4) all the ligands displayed distinctive absorption characteristics (Figure 1). The heptameric oligothiophenes, h‐FTAA and LL‐2, showed a similar absorption maximum at 434 and 437 nm, respectively. In contrast, for the heptameric ligands having a central BTD moiety, LL‐1 and LL‐3, a blue‐shifted absorption maximum (390 and 380 nm), as well as a distinct shoulder at longer wavelengths (490 nm) were observed. HS‐169, which has a similar D–A–D type electronic structure, displayed two absorption maxima (Figure 1 A) and the two absorption bands, a high energy band around 360 nm followed by a low energy band at 515 nm, likely arise from the π–π* transition and charge‐transfer transition, respectively. Thus, the latter is less pronounced for the heptameric LL‐1 and LL‐3 compared to the pentameric HS‐169.

### Optical characterization of LL‐1 and LL‐3 bound to recombinant Aβ 1–42 fibrils

**Figure 2:**
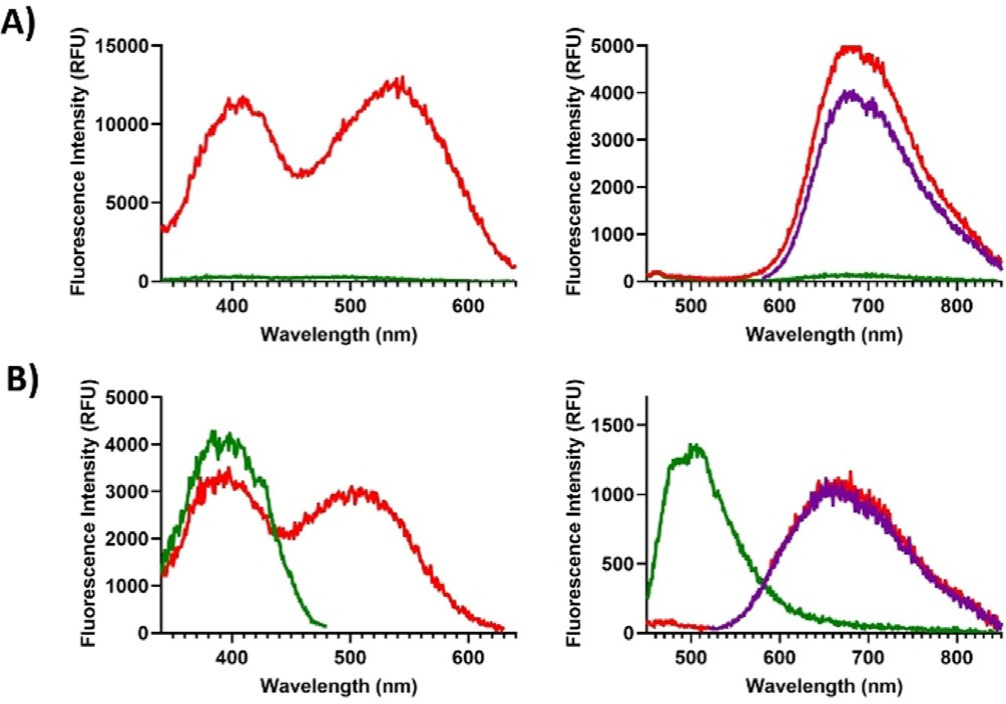
Excitation‐ (left) and emission (right) spectra of 600 nm LL‐1 (A) and LL‐3 (B), in PBS pH 7.4 (green spectra) or mixed with 10 μm recombinant Aβ 1–42 amyloid‐like fibrils (red or purple spectra). The purple and red emission spectra correlate to excitation at the first (390 or 410 nm) or second (510 or 540 nm) excitation maxima, respectively. The excitation spectra were collected for the respective emission maxima.

HS‐169 and heptameric oligothiophenes, including h‐FTAA, have been shown to exhibit specific spectral signatures when bound to protein aggregates.^19^, ^22^, ^30^, ^34^, ^36^, ^37^ To elucidate selective binding of the novel heptameric thiophene‐based D–A–D ligands to Aβ aggregates, LL‐1 and LL‐3 were next tested towards thioflavin (ThT)^5^, ^38^ and HS‐169 positive amyloid‐like fibrils (Supporting Information, Figure S1) made from recombinant Aβ 1–42 peptide. Both ligands revealed distinct excitation and emission characteristics when bound to recombinant Aβ fibrils (Figure 2). When mixed with amyloid‐like Aβ 1–42 fibrils, LL‐1 showed a remarkable enhancement of both the excitation and emission intensity (Figure 2 A). Thus, similar to the most commonly used amyloid‐specific dye, ThT,^5^, ^38^ LL‐1 revealed a strong increase in fluorescence upon binding to amyloid fibrils. In addition, LL‐1 displayed a red‐shifted excitation when bound to Aβ 1–42 amyloid‐like fibrils (Supporting Information, Figure S1).

For LL‐3, the spectral difference between free ligand or the ligand bound to recombinant amyloid‐like Aβ 1–42 fibrils was even more evident. In PBS, LL‐3 showed an emission maximum around 500 nm, whereas bound to Aβ 1–42 fibrils, the ligand exhibited a red‐shifted spectrum with an emission maximum around 680 nm (Figure 2 B). This phenomenon was also observed in excitation mode, since the spectrum for LL‐3 bound to fibrils showed an additional peak around 510 nm compared to the spectra for the ligand in PBS (Figure 2 B). The excitation‐ and emission spectrum for LL‐3 in PBS resemble the fluorescence characteristics observed from pure oligothiophenes,^23^, ^29^, ^30^, ^31^ suggesting that the intramolecular charge transfer transition to the BTD motif is restricted for the free ligand in PBS. On the other hand, this intramolecular charge transfer probably occurs when LL‐3 binds to Aβ 1–42 fibrils, since the optical characteristics resemble the excitation‐ and emission spectrum for LL‐1 (Figure 2 A), as well as HS‐169 bound to Aβ 1–42 fibrils.^34^ Hence, LL‐3 is most likely adopting a strikingly different conformation bound to Aβ 1–42 fibrils compared to unbound ligand in PBS. Further photophysical studies and theoretical calculations are necessary to resolve this matter in more detail. Overall, we conclude that both ligands provided distinct optical signatures upon binding to Aβ 1–42 fibrils, verifying that these ligands could be utilized for fluorescent assignment of recombinant amyloid‐like fibrils.

### Optical characterization of LL‐1 and LL‐3 bound to Aβ deposits in brain tissue sections from transgenic mice

Several studies have shown that thiophene‐based ligands identify a broader subset of disease‐associated protein aggregates than conventional amyloid ligands.^23^, ^29^, ^30^, ^31^ For instance, two types of Aβ deposits, Aβ core plaques in the brain parenchyma and cerebral β‐amyloid angiopathy (CAA) in the vasculature, have been selectively identified by oligothiophenes in brain tissue sections.^29^, ^30^, ^31^, ^34^ Therefore, the ligands were next evaluated towards brain tissue sections from APP23 transgenic mice with AD‐like pathology.

**Figure 3:**
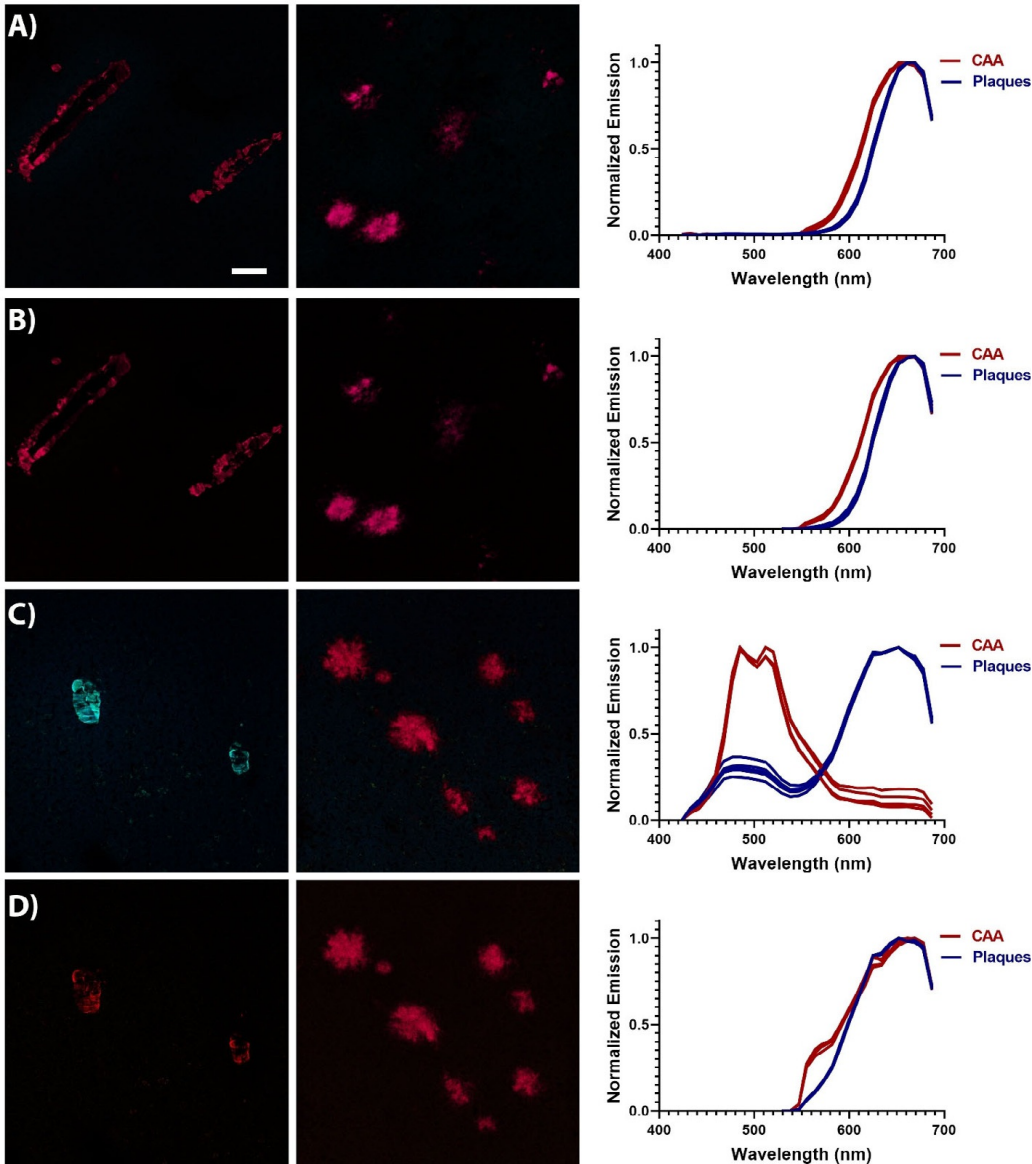
Optical characterization of LL‐1 and LL‐3 bound to Aβ deposits in brain tissue section from APP23 transgenic mice: A,B) Images of LL‐1 labelled CAA (left) and Aβ core plaques (middle), as well as emission spectra (right) for LL‐1 bound to the different Aβ deposits upon excitation at 405 nm (A) or 535 nm (B). C,D) Images of LL‐3 labelled CAA (left) and Aβ core plaques (middle), as well as emission spectra (right) for LL‐3 bound to the different Aβ deposits upon excitation at 405 nm (C) or 535 nm (D). Spectra were collected from 10 individual deposits. Scale bar represents 50 μm.

All the heptameric ligands, as well as HS‐169, showed selective binding to Aβ core plaques and CAA (Figure 3 and Supporting Information, Figure S2 and S3). As previously reported,^30^ h‐FTAA displayed well‐resolved emission spectra with characteristic double peaks upon binding to both Aβ assemblies and the novel heptameric oligothiophene, LL‐2, showed similar spectral characteristics with slightly less defined double peaks (Supporting Information, Figure S2). HS‐169 displayed similar emission spectra as reported for the ligand bound to Aβ deposits in human brain tissue sections with AD pathology.^34^ When bound to Aβ core plaques, an emission profile with a maximum around 675 nm was obtained, whereas the emission spectrum was slightly red‐shifted from the ligand bound to CAA (SI, Figure S2).

When stained in combination with an antibody (4G8) towards Aβ pathology, the novel heptameric D–A–D ligands showed good correlation with the antibody staining (Supporting Information, Figure S3). Thus, LL‐1 and LL‐3 selectively identified immunopositive aggregated Aβ species. Similar to the observation on recombinant Aβ 1–42 amyloid‐like fibrils, upon excitation at 405 nm or 535 nm, LL‐1 showed a broad fluorescence spectrum with an emission maximum close to 700 nm when bound to Aβ deposits in brain tissue sections (Figure 3 A and 3B). Moreover, the spectra from CAA were slightly blue‐shifted compared to the emission spectrum obtained from Aβ core plaques. In contrast, LL‐3 showed two distinct emission profiles bound to CAA or Aβ core plaques (Figure 3 C,D). Upon excitation at 405 nm, LL‐3 bound to Aβ core plaques displayed an emission profile with a strong emission peak around 675 nm and a weaker emission peak around 500 nm, whereas a strikingly different emission spectrum (*λ*_max_ around 500 nm) was observed for LL‐3 bound to CAA (Figure 3 C). Thus, the spectral signatures from LL‐3 could be utilized to distinguish these two aggregated Aβ species. Analogous to the observation for LL‐3 free in PBS or bound to recombinant A*β* 1–42 amyloid‐like fibrils (Figure 2 B), the ligand is presumably binding in different modes to CAA or Aβ core plaques, which renders distinct photophysical properties and specific emission profiles.

From a chemical perspective, these experiments supported that thiophene‐based heptameric ligands having the central thiophene unit replaced with a BTD moiety could selectively detect CAA or Aβ core plaques in brain tissue sections from APP23 mice. Secondly, LL‐3 gave a better spectral separation of these aggregated Aβ species than LL‐1, suggesting that the amount of carboxylic acid side chain functionalities, as well as their spacing along the conjugated backbone are crucial chemical determinants for achieving superior ligands for assigning distinct Aβ species. These chemical determinants have also been essential for obtaining improved tetrameric oligothiophenes for spectral separation of age‐related Aβ and tau aggregates,^23^ as well as for achieving pentameric oligothiophenes exhibiting a greater therapeutic effect in prion infected mice.^39^ Furthermore, when using a combination of a tetrameric LCO and h‐FTAA (Figure 1 B), the spectra from the cores of Aβ plaques differed significantly among familial and sporadic AD subtypes.^19^ However, previously reported LCOs have not been able to distinguish CAA and Aβ core plaques as efficiently as LL‐3. Therefore, it would be of great interest to evaluate the novel D–A–D heptameric ligands, independently or in combination with other LCOs, towards brain tissue samples from different cases with familial or sporadic AD and such studies are ongoing.

### Synthesis and characterization of an additional thiophene‐based D–A–D heptameric ligand

**Scheme 2:**
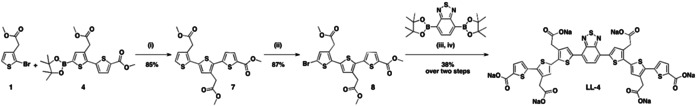
Synthesis of LL‐4: Reagents and conditions: (i) PEPPSI™‐IPr, K_2_CO_3_, toluene/MeOH (1:1), 80 °C, 30 min; (ii) chloroform, −15 °C to r.t., 4.5 h; (iii) PEPPSI™‐IPr, K_2_CO_3_, toluene/MeOH (1:1), MW 100 °C, 30 min; (iv) NaOH (1 m, aq.), 1,4‐dioxane, 40 °C.

**Figure 4:**
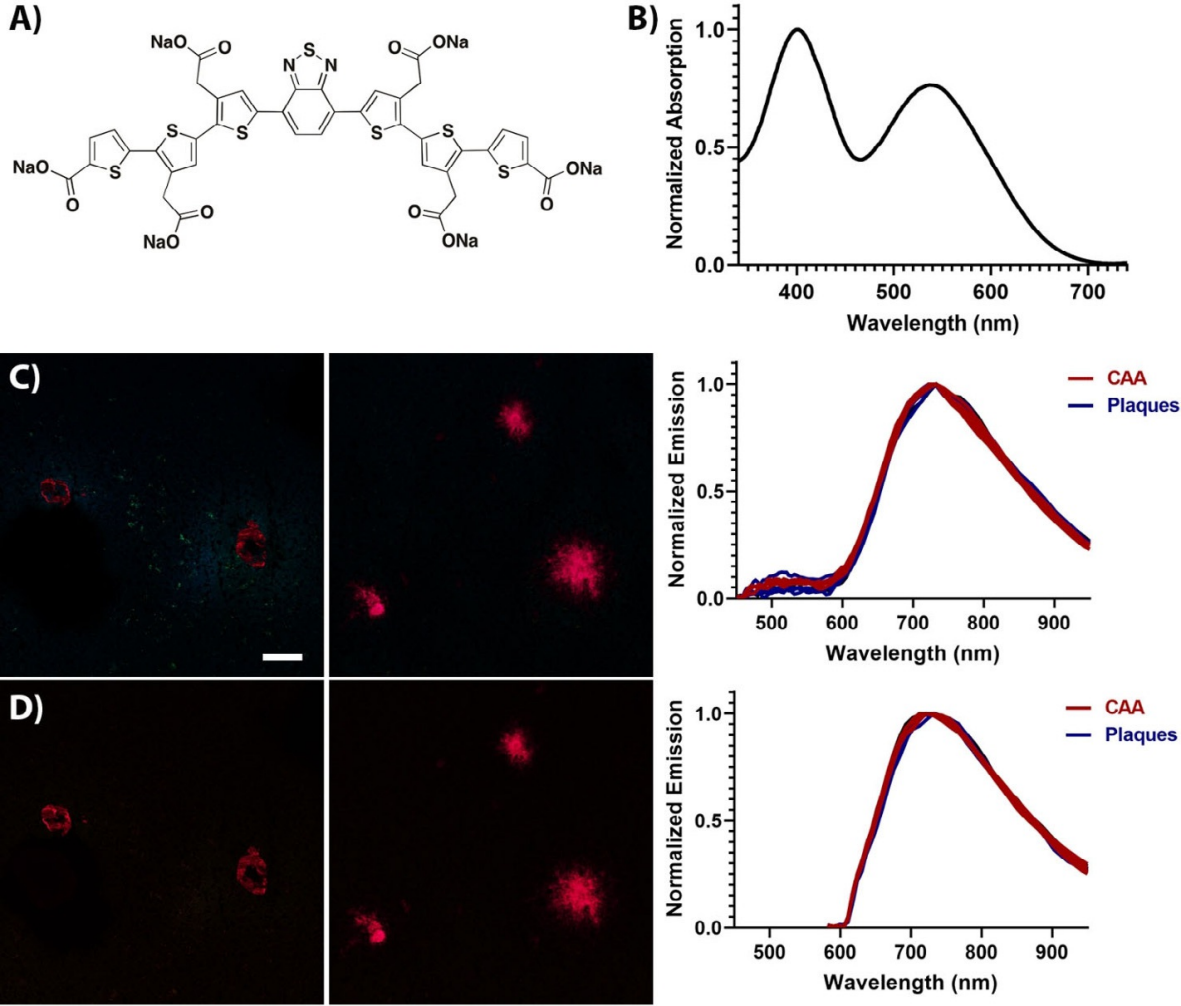
Chemical structure and optical characterization of LL‐4: A) Chemical structure of LL‐4. B) Absorption spectrum of 30 μm LL‐4 in PBS pH 7.4. C,D) Images of LL‐4 labelled CAA (left) and Aβ core plaques (middle), as well as emission spectra (right) for LL‐4 bound to the different Aβ deposits upon excitation at 405 nm (C) or 535 nm (D). Spectra were collected from 10 individual deposits. Scale bar represents 50 μm.

To elucidate the importance of the amount of carboxylic acid side chain functionalities, as well as their spacing along the conjugated backbone, we synthesized an additional thiophene‐based D–A–D heptameric ligand, denoted LL‐4 (Scheme 2 and Figure 4 A). LL‐4 was synthesized in a similar fashion as the other ligands starting with methyl ester protected 2‐bromothiophene acetic acid.^30^ By applying a palladium‐mediated Suzuki–Miyaura cross‐coupling reaction followed by bromination with NBS in sequential steps with a monoborylated bithiophene derivative^23^, ^34^ and diborylated BTD, LL‐4 was achieved in affordable yield (Scheme 2). Similar to LL‐3, LL‐4 has six carboxylate side‐chain functionalities. However, the positions of the acetic acetate side chains are altered on the thiophene rings adjacent to the central BTD moiety. Thus, LL‐4 is an isomer to LL‐3 having the acetate side chains of the central trimeric thiophene‐BTD‐thiophene unit tail‐to‐tail instead of head‐to‐head.

When diluted in PBS, LL‐4 showed a distinctive absorption spectrum with two absorption maxima at 400 and 545 nm (Figure 4 B). As mentioned earlier, these absorption bands likely arise from the π–π* transition and charge‐transfer transition, respectively, and the latter band was much more pronounced for LL‐4 compared to LL‐1 and LL‐3 (Figure 1 C,E). Instead, the spectrum resembled the optical signature from HS‐169 (Figure 1 A). Hence, the charge‐transfer transition seems to be more favorable for ligands having the acetate side chains of the central trimeric thiophene‐BTD‐thiophene unit tail‐to‐tail (HS‐169 and LL‐4) instead of head‐to‐head (LL‐1 and LL‐3).

LL‐4 was next evaluated towards brain tissue sections from APP23 transgenic mice with AD‐like pathology. The ligand revealed selective binding to both Aβ core plaques and CAA (Figure 4 C and 4D). The emission spectra from LL‐4 were similar for both aggregated Aβ species with emission maxima around 730 nm. Thus, in contrast to LL‐3, LL‐4 lacked the ability to distinguish Aβ core plaques from CAA, suggesting that a distinct periodicity of carboxylic groups along the heptameric backbone is necessary to achieve a ligand that can differentiate these aggregated Aβ entities. Overall, the characterization experiments confirmed that the two isomers, LL‐3 and LL‐4, displayed different photophysical characteristics, both in solution and bound to Aβ aggregates.

### pH‐dependent optical characteristics of LL‐1, LL‐3 and LL‐4

**Figure 5:**
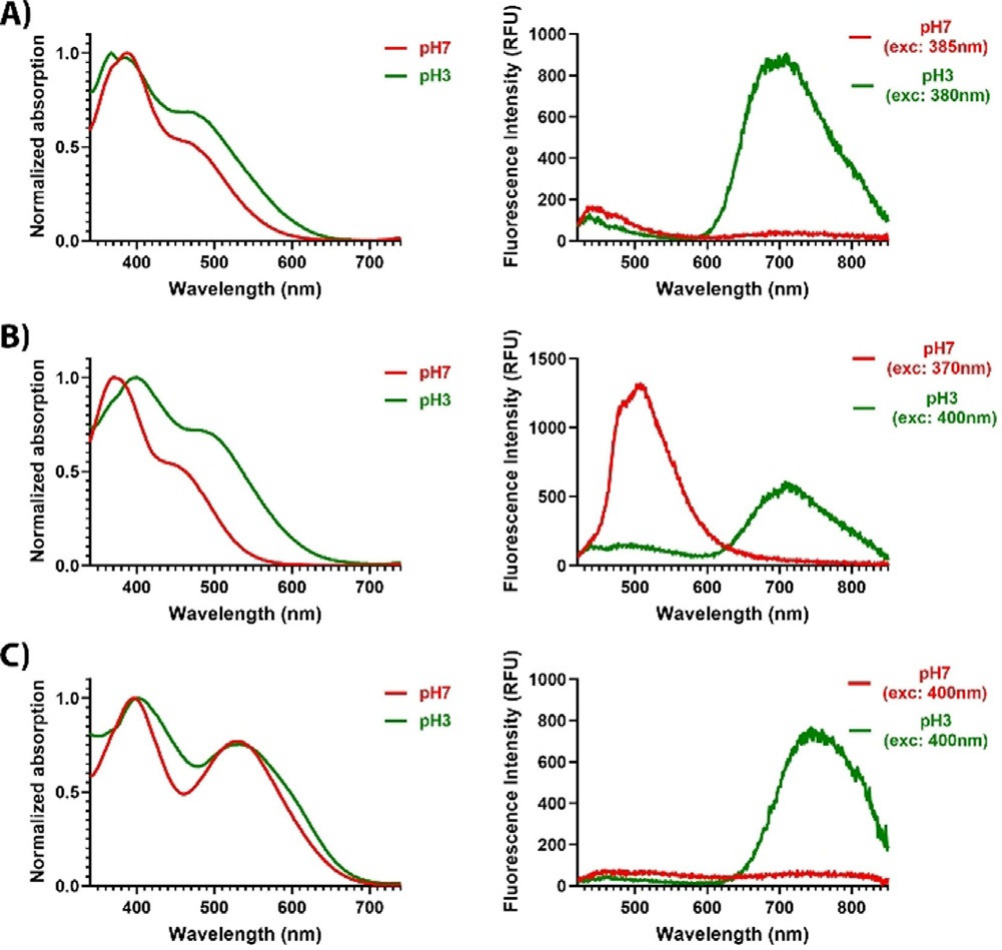
pH‐dependent optical characteristics of LL‐1, LL‐3 and LL‐4: A) Absorption‐ and emission spectra of 30 μm LL‐1 in 20 mm Na‐phosphate pH 7 (red) or 20 mm Na‐citrate pH 3 (green). B) Absorption‐ and emission spectra of 30 μm LL‐3 in 20 mm Na‐phosphate pH 7 (red) or 20 mm Na‐citrate pH 3 (green). C) Absorption‐ and emission spectra of 30 μm LL‐4 in 20 mm Na‐phosphate pH 7 (red) or 20 mm Na‐citrate pH 3 (green). The emission spectra were recorded with excitation corresponding to the absorption maxima with highest energy.

To clarify the observed emission characteristics of the thiophene‐based D–A–D heptameric ligands when interacting with aggregated Aβ morphotypes, we next explored solvent induced photophysical properties of the ligands. As it has previously been reported that changes in pH can mimic the optical behavior of anionic LCOs bound to different Aβ and tau morphotypes,^23^ absorption‐ and emission spectra were recorded for LL‐1, LL‐3 and LL‐4 in buffer solution with pH 3 or pH 7 (Figure 5). For LL‐1, decreasing pH induced a slight increase of the low energy absorption band around 490 nm, suggesting that the charge‐transfer transition seems to be more favorable at acidic pH (Figure 5 A). In addition, at pH 3, a strong increase of the emission intensity around 700 nm was observed. Hence, by changing the pH and thereby the charge of the carboxyl groups, the emission profile observed for LL‐1 bound to Aβ aggregates could be largely mimicked.

LL‐3 showed even more striking pH‐dependent photophysical transitions than LL‐1. Upon protonation of the carboxylates, the absorption spectrum was red‐shifted and the low energy absorption band at longer wavelengths became more pronounced (Figure 5 B). Excitation of LL‐3 at a wavelength corresponding to the absorption maxima with highest energy, resulted in two completely different emission profiles (Figure 5 B). At pH 7, LL‐3 displayed an emission spectrum with a maximum intensity around 510 nm, whereas at pH 3, dominant emission around 710 nm was detected. Thus, the two different emission profiles displayed from LL‐3 bound to Aβ core plaques or CAA in tissue sections (Figure 3 C) could be recreated in solution. The LL‐3 emission characteristics associated with CAA in APP23 transgenic mice could be obtained upon deprotonation of the carboxyl groups. Likewise, the spectral features obtained in acid conditions, when the anionic side chains were protonated, resembled the emission spectra acquired from LL‐3 bound to Aβ core plaques.

The third ligand, LL‐4, revealed similar pH‐dependent photophysical transitions as LL‐1 (Figure 5 C). The absorption spectrum at pH 3 or 7 were comparable, but under acidic conditions LL‐4 displayed a similar emission profile as obtained for the ligand bound to Aβ aggregates with an enhanced emission around 750 nm. Overall, the photophysical characterization of the ligands at different pH verified that similar spectral transitions to the ones observed for the ligands bound to Aβ deposits could be induced by altering the charge of the anionic side chain functionalities along the conjugated backbone. Moreover, these experiments also confirmed that minor alterations of the amount and periodicity of anionic groups along the heptameric backbone highly influenced the pH‐dependent optical characteristics of the ligands. Thus, similar chemical determinants that seemed important for achieving superior ligands for assigning distinct Aβ species, could be correlated to distinct pH‐dependent photophysical transitions. Additional advanced photophysical studies and theoretical calculations will be required to clarify these issues in more detail.

## Conclusions

In conclusion, thiophene‐based D–A–D heptameric ligands were identified as optical ligands for spectral assignment of Aβ aggregates. The spectral signature from one ligand could also be utilized to distinguish different Aβ morphotypes and the superior functionality of this ligand compared to structurally related compounds could be assigned to a distinct periodicity and number of carboxy substituents along the conjugated backbone. We foresee that our findings will aid in the chemical design of ligands that can be utilized for exploring different aggregated morphotypes composed of Aβ, as well as other polymorphic protein aggregates that are observed in neurodegenerative protein aggregation disorders.

## Experimental Section

Full experimental details including additional characterization data and NMR spectra of new compounds, as well as supporting figures are given in the Supporting Information.

## Conflict of interest

The authors declare no conflict of interest.

## Supporting information

As a service to our authors and readers, this journal provides supporting information supplied by the authors. Such materials are peer reviewed and may be re‐organized for online delivery, but are not copy‐edited or typeset. Technical support issues arising from supporting information (other than missing files) should be addressed to the authors.

Supplementary
