# Supplementary material for: Synthesis and Characterization of Thiophene‐based Donor–Acceptor–Donor Heptameric Ligands for Spectral Assignment of Polymorphic Amyloid‐β Deposits
Source: Chemistry. 2020 May 15;26(33):7425–32. doi: 10.1002/chem.201905612 (PMC7318160; doi:10.1002/chem.201905612)
Supplement: Supplementary file 1 — Supplementary [file CHEM-26-7425-s001.pdf]

Chemistry–A European Journal

Supporting Information

Synthesis and Characterization of Thiophene-based Donor–Acceptor–Donor Heptameric Ligands for Spectral Assignment of Polymorphic Amyloid-β Deposits

Linda Lantz, Hamid Shirani, Therése Klingstedt, and K. Peter R. Nilsson*[a]

Supporting Information

Synthesis and characterization of thiophene-based donor-acceptor-donor heptameric ligands for spectral assignment of polymorphic amyloid-β deposits

L. Lantz, H. Shirani, T. Klingstedt and K. P. R. Nilsson[*]

Division of Chemistry, Department of Physics, Chemistry and Biology, Linköping University, Linköping, Sweden.
*Corresponding author; E-mail: petni@ifm.liu.se

Table of Content

Supporting Figures S2

Experimental Details S5

References S13

NMR spectra S15

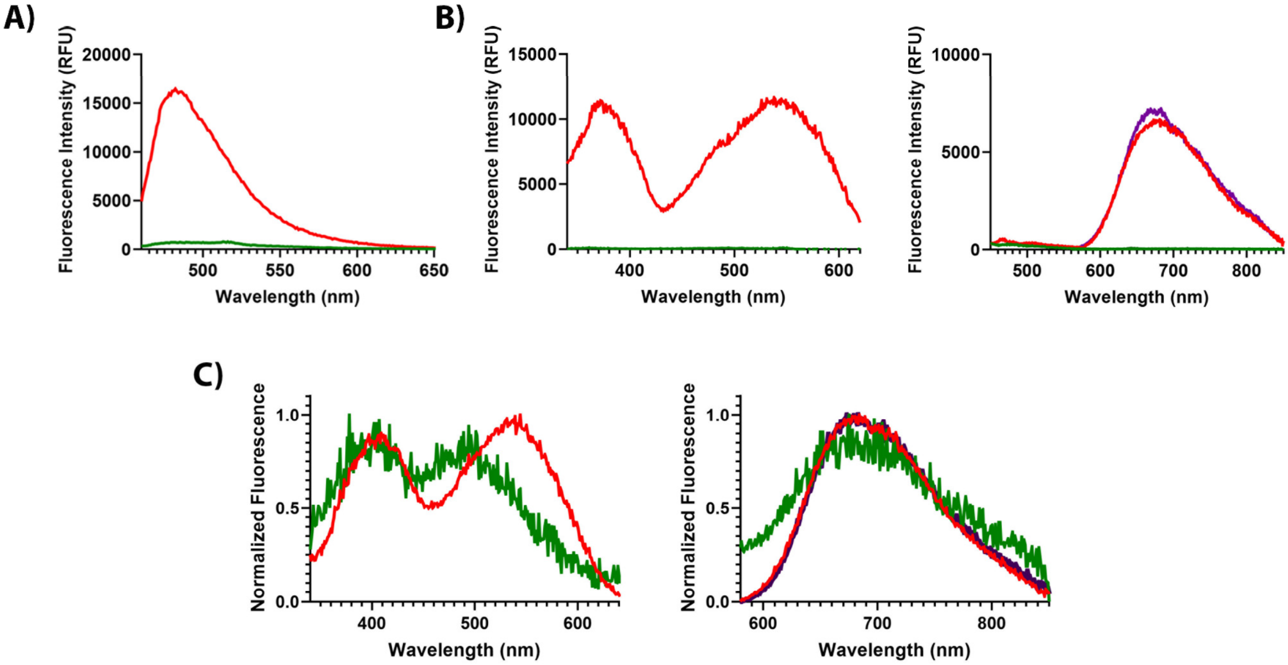

Figure S1. A) Emission spectra for 2 μM ThT in PBS pH 7.4 (green spectrum) or mixed with 10 μM recombinant Aβ 1-42 amyloid-like fibrils (red spectrum) **B)** Excitation- (left) and emission (right) spectra of 600 nM HS-169 in PBS pH 7.4 (green spectra) or mixed with 10 μM recombinant Aβ 1-42 amyloid-like fibrils (red or purple spectra). The purple and red emission spectra correlates to excitation at the first (380 nm) or second (540 nm) excitation maxima, respectively. The excitation spectra were collected for the emission maxima at 680 nm. **C)** Normalized excitation- (left) and emission (right) spectra of 600 nM LL-1 in PBS pH 7.4 (green spectra) or mixed with 10 μM recombinant Aβ 1-42 amyloid-like fibrils (red or purple spectra). The purple and red emission spectra correlate to excitation at the first (410 nm) or second (540 nm) excitation maxima, respectively.

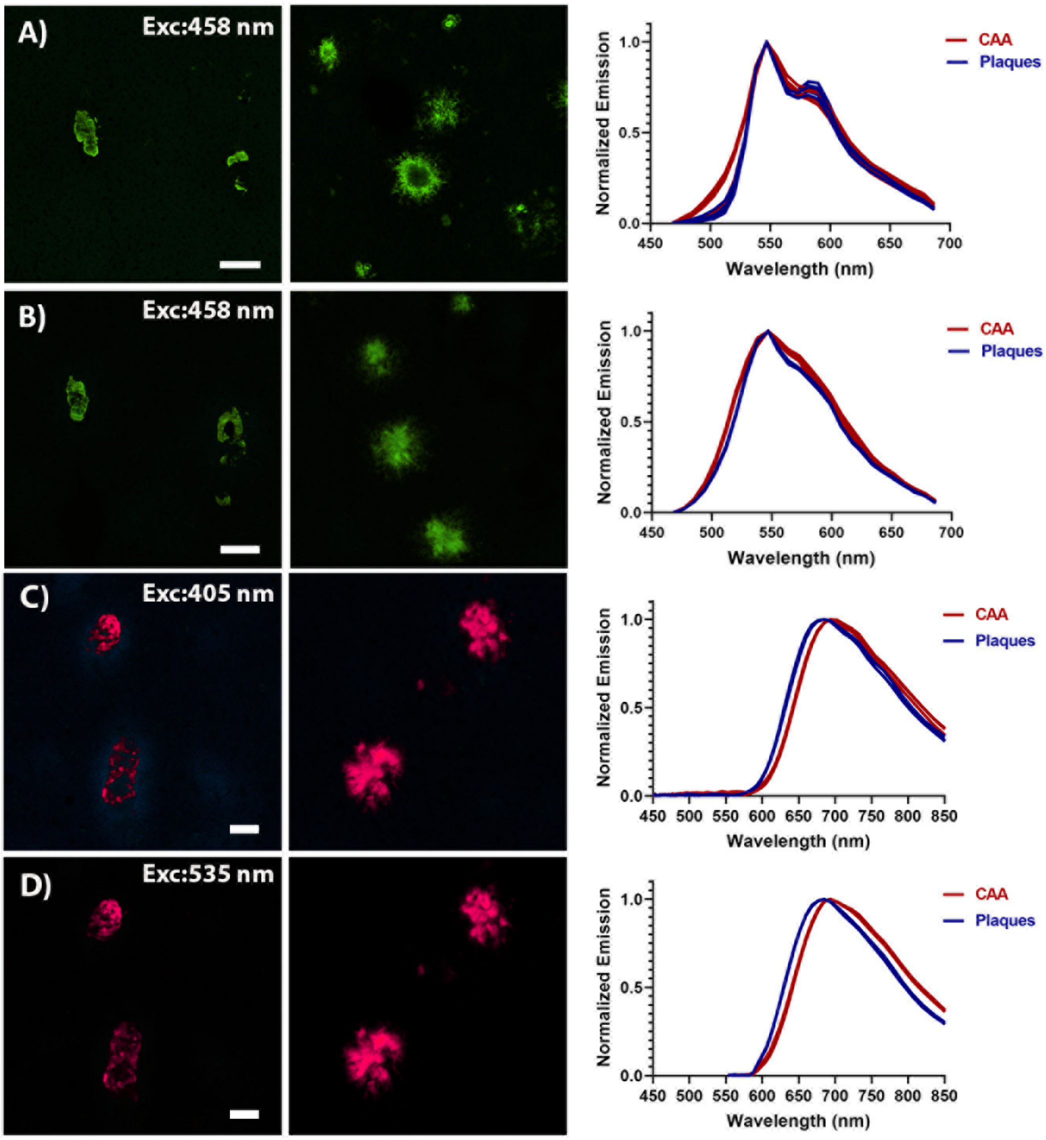

Figure S2. Fluorescence images and emission spectra of h-FTAA (**A**), LL-2 (**B**) and HS-169 (**C** and **D**) bound to Aβ deposits in the vasculature (left, CAA) or brain parenchyma (right, core plaques) in brain tissue sections from APP23 transgenic mice. The emission spectra were recorded with an excitation at 458 nm (**A** and **B**), 405 nm (**C**) or 535 nm (**D**). Scale bar represent 50 μm (**A** and **B**) or 20 μm (**C** and **D**).

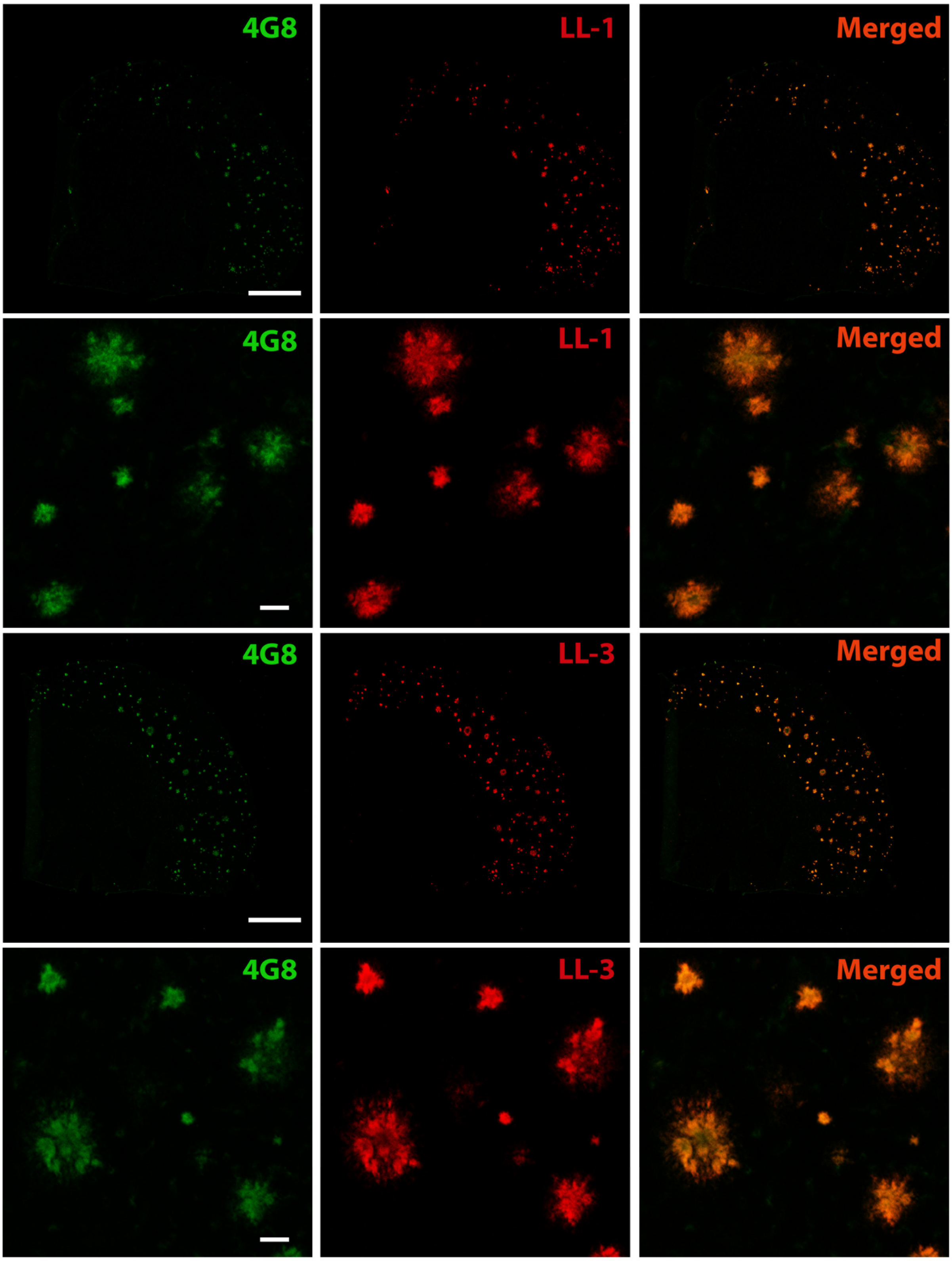

Figure S3. Fluorescence images of brain tissue sections from APP23 transgenic mice with Aβ pathology co-stained with the 4G8 anti-amyloid-β antibody and the LL-1 or LL-3 ligand. The following excitation/emission wavelengths were used to visualize the antibody and the ligand staining: 595/600-640 (Alexa Fluor 594) and 535/680-760 (LL-1 and LL-3). Scale bars represent 1 000 μm (row 1 and 3) or 50 μm (row 2 and 4).

Experimental Details

Synthesis of ligands

The synthesis of compound **1**, compound **2A-6A**, compound **4**, **HS-169** and **h-FTAA** has been published elsewhere.[1-7] The other precursor molecules and ligands were synthesized as outlined below.

General methods

Reagents and solvents were purchased from Sigma Aldrich, Merck KGaA. Flash column chromatography was performed using silica gel (60 Å, 0.040-0.063 mm, 230-400 mesh particle size, Sigma Aldrich, Merck KGaA). NMR spectra were recorded on Varian instruments (^{1}H 300 MHz, ^{13}C 75.4 MHz) or (^{1}H 500 MHz, ^{13}C 126 MHz) and chemical shifts were designated on the ppm scale using residual undeuterated solvent peak as reference. Microwave-assisted reactions were carried out on a Biotage® Initiator instrument (2.45 GHz**)**. Analytical thin-layer chromatography (TLC) was performed using silica gel 60 F_{254} glass plates (Supelco/ Sigma Aldrich, Merck KGaA), developed in an appropriate mobile phase, visualized by UV-light λ=254 nm and 366 nm, stained with an ethanolic *p*-anisaldehyde – sulfuric acid solution (ethanol/ sulfuric acid/ *p*-anisaldehyde/ acetic acid 90:3:2:1) followed by heating for visualization.

Analytical high-performance liquid chromatography (HPLC) was performed on a Waters system comprising a Waters 1525 gradient pump, 2998 Photodiode Array Detector, 2424 Evaporative Light Scattering Detector, SQD 2 Mass Detector and a Waters XBridge™ C18 column (4.6 × 50 mm, 3.5 µm). Flow rate 1 mL/min. Eluent A: 95:5 H_2O/ acetonitrile with NH_4OAC (10 mM). Eluent B: 90:10 acetonitrile/ H_2O with NH_4OAc (10 mM).

Semi-preparative HPLC was performed on a Waters system composed of a 2535 quaternary gradient pump, 2998 Photodiode Array Detector, 2424 Evaporative Light Scattering Detector, SQ Detector 2 Mass Detector, 2767 Sample Manager and a Waters XBridge™ Prep C18 column (19×250 m, 5 µm OBD™). Flow rate 25 mL/min. Eluent A: 95:5 H_2O/ acetonitrile with NH_4OAc (10 mM). Eluent B: 90:10 acetonitrile/ H_2O with NH_4OAC (10 mM).

General procedure Suzuki-Miyaura cross-coupling

The bromide (1-2 equiv.), the aryl boronic acid or boronic ester (1-2 equiv.), K_2CO_3 (3 equiv./bromide) and PEPPSI™-IPr (3 mol%/bromide) were dissolved in the below specified solvent (10 mL/ mmol). The mixture was heated to 80°C for 2h. After cooling to room temperature, the mixture was acidified with acetic acid (AcOH) (concd.), diluted with DCM and washed with 2 × 1M HCl(aq.), H_2O (DI), 2 × sat. $NaHCO_3$ (aq.) and sat. NaCl (aq.). The organic phase was separated, dried with $MgSO_4$, filtered and solvents evaporated *in vacuo* at 40°C.

General procedure bromination

To a stirred solution of the arylic compound in the below specified solvent (6 mL/ mmol) at -15°C was portion wise added a suspension of NBS (1.1 eq/ α-position, 2 mL/mmol) in DMF or chloroform. The reaction mixture was let to attain room temperature and stirred for 18h. The reaction mixture was diluted with DCM, washed with 2 × 1M HCl (aq.) and 2 × H_2O (DI). The organic phase was separated, dried with $MgSO_4$, filtered and concentrated *in vacuo* at 40°C.

General procedure methyl ester hydrolysis

To a stirred solution of the methyl ester compound in 1,4-dioxane at 40°C was added 1M NaOH (aq.)(2 equiv./ester) drop wise over a minute. Upon precipitation, H_2O (DI) was added drop wise. Extracting µl aliquots analyzed by HPLC-MS and TLC monitored the hydrolysis. After complete hydrolysis the solution was lyophilized to give the freeze-dried product as a sodium salt.

Compound 2B

2,2'-(2,2'-(benzo[*c*][1,2,5]thiadiazole-4,7-diyl)bis(thiophene-3,2-diyl))diacetic acid

The bromide **1** (0.757 g, 3.22 mmol) together with 2,1,3-benzothiadiazole-4,7-bis(boronic acid pinacol ester) (0.500 mg, 1.29 mmol) were set for general procedure of Suzuki-Miyaura cross-coupling with 1,4-dioxane/ MeOH/ toluene (1:1:1) as solvent. Purification by flash column chromatography in DCM/MeOH 400:1 gave compound **2B** (0.436 g, 76%) as a yellow solid.

^{1}H NMR (300 MHz, $CDCl_3$) δ 7.76 (s, 2H), 7.48 (d, J = 5.2 Hz, 2H), 7.21 (d, J = 5.2 Hz, 2H), 3.71 (s, 4H), 3.68 (s, 6H). ^{13}C NMR (75 MHz, $CDCl_3$) δ 171.7, 154.1, 135.1, 132.4, 130.4, 130.1, 127.0,

126.6, 52.2, 35.2. MS: *m/z* calcd for $C_{20}H_{16}N_2O_4S_3$ $(M+H)^+$: 445.04. Found: 445.5. R_f = 0.44 in DCM/ MeOH 100:1.

Compound 3B

2,2'-(2,2'-(benzo[*c*][1,2,5]thiadiazole-4,7-diyl)bis(5-bromothiophene-3,2-diyl))diacetic acid

Compound **2B** (0.334 g, 0.751 mmol) was dibrominated with NBS (0.308 g, 1.73 mmol) according to the general procedure for bromination using dry DMF/chloroform (1:1) as solvent. Purification by flash column chromatography with DCM/MeOH 400:1 as eluent gave compound **3B** (0.442 g, 97%) as a yellow solid.

^1H NMR (300 MHz, $CDCl_3$) δ 7.73 (s, 2H), 7.18 (s, 2H), 3.70 (s, 6H), 3.64 (s, 4H). ^{13}C NMR (75 MHz, $CDCl_3$) δ 171.1, 153.7, 136.4, 133.0, 132.8, 130.2, 126.2, 114.0, 52.3, 35.1. MS: *m/z* calcd for $C_{20}H_{14}Br_2N_2O_4S_3$ $(M+H)^+$: 602.85. Found: 603.00. R_f = 0.46 in DCM/ MeOH 100:1.

LL-3 methyl ester

The dibromide **3B** (0.086 g, 0.143 mmol) and previously synthesized compound **4**[4](0.133 g, 0.314 mmol) were reacted according to the general Suzuki-Miyaura cross-coupling with 1,4-dioxane as solvent. Flash column chromatography purification using DCM/MeOH (100:1) as eluent gave **LL-3 methyl ester** (0.126 g, 85%) as dark brown-purple solid.

^1H NMR (300 MHz, $CDCl_3$) δ 7.81 (s, 2H), 7.76 (d, *J* = 3.9 Hz, 2H), 7.31 (s, 2H), 7.21-7.22 (m, 4H), 3.91 (s, 6H), 3.80 (s, 4H), 3.76 (s, 6H), 3.73 (s, 6H), 3.72 (s, 4H). ^{13}C NMR (75 MHz, $CDCl_3$) δ 171.4, 170.8, 162.5, 153.8, 141.8, 137.3, 136.6, 134.7, 134.2, 133.4, 133.3, 132.2, 131.8, 130.1, 127.6, 127.3, 127.1, 126.5, 52.5, 52.41, 52.37, 35.4, 35.0. MS: *m/z* calcd for $C_{46}H_{36}N_2O_{12}S_7$ $(M+H)^+$: 1033.04. Found: 1033.74. R_f = 0.09 in DCM/ MeOH 100:1.

LL-3

The hexamethyl ester **LL-3 methyl ester** was treated according to the general procedure of methyl ester hydrolysis. Lyophilization gave **LL-3** as blood red solid sodium salt.

^1H NMR (500 MHz, D_2O) δ 7.48 (s, 2H), 7.39 (d, *J* = 3.8 Hz, 2H), 7.14 (s, 2H), 7.01 (s, 2H), 6.99 (d, *J* = 3.9 Hz, 2H), 3.64 (s, 4H), 3.59 (s, 4H). ^{13}C NMR (126 MHz, D_2O) δ 179.6, 179.2, 169.6, 152.5, 139.9, 139.6, 136.8, 136.6, 134.9, 134.8, 132.4, 131.3, 131.2, 129.0, 128.2, 127.3, 125.6, 125.0, 38.6, 38.3. MS: *m/z* calcd for $C_{40}H_{24}N_2O_{12}S_7$ $(M+H)^+$: 948.94. Found: 949.32.

LL-2 methyl ester

The general Suzuki-Miyaura cross-coupling reaction was applied on dibromide **3A** (0.100 g, 0.182 mmol) and boronic ester **4**[4] (0.169 g, 0.400 mmol) in 1,4-dioxane. The solid crude product was washed with warm methanol. Purification by flash column chromatography was performed twice using DCM/MeOH (50:1) respectively DCM/MeOH (100:1) as eluents, this gave **LL-2 methyl ester** (0.108 g, 61%) as red solid.

^{1}H NMR (300 MHz, $CDCl_3$) δ 7.74 (d, *J* = 3.9 Hz, 2H), 7.20–7.16 (m, 4H), 7.14 (s, 2H), 7.13 (s, 2H), 3.89 (s, 6H), 3.77 (bs, 8H), 3.76 (s, 6H), 3.75 (s, 6H). ^{13}C NMR (75 MHz, $CDCl_3$) δ 171.0, 170.8, 162.4, 141.7, 136.4, 135.5, 135.1, 134.2, 133.2, 132.5, 132.2, 131.6, 131.2, 127.6, 127.4, 127.0, 52.52, 52.48, 52.39, 34.9. MS: *m/z* calcd for $C_{44}H_{36}O_{12}S_7$ $(M+NH_4)^+$: 998.06. Found: 998.60. R_f = 0.26 in DCM/ MeOH 50:1.

LL-2

The general procedure for methyl ester hydrolysis was applied on **LL-2 methyl ester**. Lyophilization gave the sodium salt of **LL-2** as red-orange solid.

^{1}H NMR (500 MHz, D_2O) δ 7.26 (s, 2H), 6.93 (s, 2H), 6.92 (s, 2H), 6.83 (s, 2H), 6.76 (s, 2H), 3.56 (s, 4H), 3.48 (s, 4H). ^{13}C NMR (126 MHz, D_2O) δ 179.2, 179.1, 169.6, 139.8, 139.4, 135.1, 134.8, 134.6, 133.83, 133.79, 131.2, 131.1, 130.8, 127.9, 125.8, 125.5, 125.4, 38.4, 38.3. MS: *m/z* calcd for $C_{38}H_{24}O_{12}S_7$ $(M+NH_4)^+$: 913.97. Found: 914.63.

Compound 5B

PEPPSI™-IPr (2mg, 0.003 mmol) was added to a solution of dibromide **3B** (33 mg, 0.055 mmol), 2-thienylboronic acid (15 mg, 0.120 mmol) and K_2CO_3 (42 mg, 0.301 mmol) in toluene/MeOH (1:1, 2 mL) in a microwave vial. Microwave irradiation at 100°C for 20 min was followed by work-up as in the general procedure for Suzuki-Miyaura cross-coupling. Flash column chromatography with DCM/ MeOH (400:1) as eluent yielded **5B** (33 mg, 98%) as orange solid.

^{1}H NMR (300 MHz, $CDCl_3$) δ 7.80 (s, 2H), 7.29 (s, 2H), 7.27-7.25 (m, 4H), 7.06-7.02 (m, 2H), 3.72 (s, 10H). ^{13}C NMR (75 MHz, $CDCl_3$) δ 171.5, 153.9, 138.5, 136.9, 133.8, 133.2, 130.1, 128.0, 126.7, 126.6, 125.1, 124.4, 52.3, 35.4. MS: *m/z* calcd for $C_{28}H_{20}N_2O_4S_5$ $(M+H)^+$: 609.01. Found: 609.51. R_f = 0.46 in DCM/ MeOH 100:1.

Compound 6B

General procedure for bromination was applied for **5B** (33 mg, 0.054 mmol) and NBS (22 mg, 0.125 mmol) in chloroform as solvent. The dibrominated product **6B** (41mg, 98%) was isolated as tomato red solid and was characterized and used without further purification.

^{1}H NMR (300 MHz, $CDCl_3$) δ 7.79 (s, 2H), 7.22 (s, 2H), 7.00 (s, 4H), 3.72 (s, 6H), 3.70 (s, 4H). ^{13}C NMR (75 MHz, $CDCl_3$) δ 171.4, 153.9, 138.4, 137.5, 134.3, 133.3, 130.9, 130.2, 126.9, 126.5, 124.5, 111.8, 52.4, 35.4. MS: *m/z* calcd for $C_{28}H_{18}Br_2N_2O_4S_5$ $(M+H)^+$: 766.83. Found 767.01. R_f = 0.46 in DCM/ MeOH 100:1.

LL-1 methylester

The dibromide **6B** (20 mg, 0.026 mmol) together with 5-formyl-2-thienyl boronic acid (10 mg, 0.060 mmol), K_2CO_3 (21 mg, 0.151 mmol) and PEPPSI™-IPr (1.1 mg, 0.002 mmol) were dispensed in a microwave vial and dissolved in toluene/MeOH/1,4-dioxane (3 mL, 1:1:1). The reaction mixture was heated under microwave irradiation at 100°C for 40 minutes, then let to attain room temperature where after AcOH (concd.) was added until acidic pH. The mixture was diluted with ethyl acetate (250 mL), washed with 2 × 200 mL HCl (1M, aq.) and H_2O (DI). The organic phase was separated, dried over $MgSO_4$, filtered and solvent evaporated *in vacuo* to give the crude product as deep red solid. The crude product was used without further purification and was not further characterized prior hydrolysis. MS: *m/z* calcd for $C_{38}H_{24}N_2O_8S_7$ $(M+H)^+$: 860.97. Found: 861.59.

LL-1

Hydrolysis of diester **LL-1 methylester** was performed as described in the general procedure. The crude product was purified by gradient HPLC (10-50% organic phase over 6 minutes, followed by isocratic 100% organic phase for 9 min) to give **LL-1** (6 mg, 0.007 mmol, 28 % over two steps) as an ammonium salt. The ammonium salt was dissolved in 1,4-dioxane (0.6 mL) and 1M NaOH (aq.)(0.049 mL, 0.049 mmol) was added drop wise. Addition of H_2O (DI) (0.6 mL) gave a homogeneous red solution. Lyophilization gave **LL-1** as deep terracotta red sodium salt.

^{1}H NMR (300 MHz, CD_3OD) δ 8.01 (s, 2H), 7.46 (d, *J* = 3.8 Hz, 2H), 7.42 (s, 2H), 7.23 (s, 4H), 7.17 (d, *J* = 3.8 Hz, 2H), 3.60 (s, 4H). ^{13}C NMR (126 MHz, CD_3OD) δ 179.2, 169.7, 155.1, 143.5, 143.3,

139.3, 138.1, 138.0, 137.7, 134.2, 131.7, 131.2, 128.6, 127.8, 126.0, 125.5, 124.7, 40.2. MS: *m/z* calcd for $C_{36}H_{20}N_2O_8S_7$ $(M+H)^+$: 832.93. Found: 833.63.

Compound 7

Bromide **1** (73 mg, 0.313 mmol), boronic ester **4**[4] (0.110 g, 0.260 mmol), K_2CO_3 (0.130 g, 0.938 mmol) and PEPPSI™-Pr (4 mg, 0.006 mmol) was dissolved in toluene/ MeOH (1:1, 2 mL) and subjected to Suzuki-Miyaura cross-coupling by heating the mixture to 80°C for 30 minutes. The reaction mixture was let to cool to room temperature and 1M HCl was added until acidic pH. The mixture was diluted with ethyl acetate (200 mL), washed with 2 × 150 mL HCl (1M, aq.), H_2O (DI), 2 × 150 mL $NaHCO_3$ (sat., aq.) and NaCl (sat., aq.). The combined organic phase was dried over $MgSO_4$, filtered and concentrated under reduced pressure to give the crude product as yellow oil. Purification by flash column chromatography with toluene/ethyl acetate (80:1→20:1) gave **7** (0.100 g, 85%).

^{1}H NMR (300 MHz, $CDCl_3$) δ 7.74 (d, *J* = 3.9 Hz, 1H), 7.24 (d, *J* = 5.0 Hz, 1H), 7.18 (d, *J* = 3.9 Hz, 1H), 7.13 (s, 1H), 7.03 (d, *J* = 5.2 Hz, 1H), 3.89 (s, 3H), 3.78 (s, 4H), 3.73 (s, 3H), 3.72 (s, 3H). ^{13}C NMR (75 MHz, $CDCl_3$) δ 171.2, 170.8, 162.4, 141.6, 135.2, 134.1, 133.36, 133.34, 132.8, 132.3, 132.0, 131.0, 130.5, 130.1, 127.1, 125.1, 52.4, 52.3, 52.2, 34.8, 34.7. MS: *m/z* calcd for $C_{20}H_{18}O_6S_3$ $(M+H)^+$: 451.03. Found: 451.16. R_f= 0.27 in toluene/ ethyl acetate 20:1.

Compound 8

General procedure for bromination was applied on compound **7** (0.100 g, 0.222 mmol) and NBS (41 mg, 0.233 mmol) in chloroform as solvent and reaction time 4.5 hours. The reaction mixture was diluted with ethyl acetate (150 mL), washed with 2 × 100 mL HCl (1M, aq.) and 2 × 150 mL H_2O (DI). The organic phase was separated, dried with $MgSO_4$, filtered and solvent evaporated *in vacuo.* Flash column chromatography purification using toluene/ethyl acetate (20:1) as eluent gave compound **8** (0.102 g, 87%) as pale yellow solid.

^{1}H NMR (300 MHz, $CDCl_3$) δ 7.75 (d, *J* = 3.9 Hz, 1H), 7.18 (*J* = 3.9 Hz, 1H), 7.09 (s, 1H), 7.02 (s, 1H), 3.90 (s, 3H), 3.78 (s, 2H), 3.74 (s, 3H), 3.73 (s, 3H), 3.72 (s, 2H). ^{13}C NMR (75 MHz, $CDCl_3$) δ 170.82, 170.77, 162.4, 141.3, 134.1, 133.9, 133.8, 133.6, 133.4, 133.1, 132.1, 131.5, 130.5, 127.4, 112.1, 52.47, 52.46, 52.41, 34.8, 34.6. MS: *m/z* calcd for $C_{20}H_{17}BrO_6S_3$ $(M+NH_4)^+$: 547.97 Found: 548.21. R_f = 0.28 in toluene/ ethyl acetate 20:1.

LL-4 methyl ester

To a mixture of compound **8** (50 mg, 0.094 mmol), 2,1,3-benzothiadiazole-4,7-bis(boronic acid pinacol ester) (18 mg, 0.046 mmol) and K_2CO_3 (39 mg, 0.283 mmol) in toluene/MeOH (1:1, 1.25 mL) in a micro wave vial was added PEPPSI™-IPr (4 mg, 0.006 mmol). The reaction mixture was heated under microwave irradiation at 100°C for 30 minutes. The reaction mixture was let to attain room temperature and AcOH (concd.) was added until acidic pH. The mixture was diluted with DCM (250 mL) and washed with 2 × 150 mL HCl (1M, aq.), H_2O (DI) and 2 × 150 mL $NaHCO_3$ (sat., aq.). The organic layer was dried over $MgSO_4$, filtered and concentrated under reduced pressure. Purification by HPLC (isocratic 100 % organic eluent) followed by flash column chromatography using DCM/MeOH (100:1) as eluent gave hexamethyl ester **LL-4 methyl ester** (18 mg, 38 %) as aubergine coloured solid.

^{1}H NMR (300 MHz, $CDCl_3$) δ 8.05 (s, 2H), 7.84 (s, 2H), 7.77 (d, *J* = 3.9 Hz, 2H), 7.26 (s, 2H), 7.22 (d, *J* = 3.9 Hz, 2H), 3.91 (s, 6H), 3.89 (s, 4H), 3.82 (s, 4H), 3.79 (s, 6H), 3.77 (s, 6H).

^{13}C NMR (75 MHz, $CDCl_3$) δ 171.2, 170.9, 162.5, 152.5, 141.6, 138.2, 135.0, 134.2, 133.5, 133.1, 132.2, 131.6, 131.0, 130.2, 127.2, 125.50, 125.46, 52.52, 52.49, 52.43, 35.1, 35.0. MS: *m/z* calcd for $C_{46}H_{36}N_2O_{12}S_7$ $(M+H)^+$: 1033.04 Found: 1033.60. R_f = 0.26 in DCM/ MeOH 50:1.

LL-4

To a stirred solution of hexamethyl ester **LL-4 methyl ester** (18 mg, 0.017 mmol) in 1,4-dioxane (0.7 mL) at 40°C was added NaOH (1M, aq.)(0.457 mL, 0.457 mmol) drop wise over a minute. Upon precipitation H_2O (DI)(0.7 mL) was added drop wise to give a homogeneous solution. Completion of reaction was monitored by HPLC. The solution was let to attain room temperature and HCl (1M, aq.) was added until acidic pH. The reaction mixture was extracted with ethyl acetate/1,4-dioxane (3:1) and washed with H_2O (DI). The organic phase was separated, dried over $MgSO_4$, filtered and concentrated *in vacuo* to give the hexacarboxylic acid **LL-4** (17 mg, quant.) as aubergine coloured powder. Adding NaOH (1M, aq.)(1.5 equiv/ acid) to **LL-4** and diluting it with H_2O (DI) obtained the sodium salt form of **LL-4**. MS: *m/z* calcd for $C_{40}H_{24}N_2O_{12}S_7$ $(M+H)^+$: 948.95 Found: 948.92.

Optical characterization of the ligands

Stock solutions of ligands (1.5 mM in de-ionized water) were diluted to 30 μM in 20 mM Na-citrate buffer pH 3.0, 20 mM Na-acetate buffer pH 4.0 or pH 5.0, 20 mM Na-phosphate buffer pH 6.0 or pH 7.0 and phosphate buffer saline (PBS, 10 mM phosphate, 140 mM NaCl, 2.7 mM KCl, pH 7.4). Excitation- and emission spectra of the ligands were collected using an Infinite M1000 Pro microplate reader (Tecan, Männedorf, Switzerland).

Characterization of the ligands towards recombinant Aβ 1-42 amyloid fibrils

The fibrillation protocol of Aβ1-42 has been published elsewhere.[3] Briefly, recombinant Aβ1-42 peptide lyophilized in hydroxyfluoroisopropanol (rPeptide, Athens, GA, USA) was dissolved in 2 mM NaOH to a stock concentration of 1 mg/ml. The solution of Aβ1-42 was diluted with PBS to a final concentration of 10 μM and this preparation was incubated at 37°C in quiescent mode for 48 hours and the presence of recombinant Aβ1-42 amyloid fibrils was confirmed by thioflavin-T (ThT) staining. The respective ligand (600 nM) or 2 μM ThT was added to 10 μM fibrils or PBS and the excitation- and emission spectrum for each probe were collected using a Tecan Saphire^2 microplate reader (Tecan, Männedorf, Switzerland).

Staining and spectral analysis of histological samples

Brain tissue samples from transgenic APP23 mice with Aβ pathology were kindly provided by Prof. Frank Heppner, Department of Neuropathology, Charité-Universitätsmedizin Berlin, Germany, or Prof. Mathias Jucker, Hertie Institute for Clinical Brain Research, Department of Cellular Neurology, University of Tübingen, Tübingen, Germany and all animal experiments were performed in accordance to protocols approved by the local ethical committees. Frozen brain sections (20 μm thickness) from transgenic APP23 mice were fixed in 96% EtOH, rehydrated in 50% EtOH and de-ionized water followed by incubation in PBS for 10 min. Stock solutions (1.5 mM ligand in de-ionized water) of HS-169 and the heptameric ligands, h-FTAA, LL-1, LL-2, LL-3 and LL-4 were diluted to 600 nM in PBS and added to the sections. After 30 min, the sections were washed with PBS and mounted with Dako fluorescent mounting medium (Dako Cytomation, Glostrup, Denmark). The mounting medium was allowed to solidify over night before collecting emission spectra of the ligands bound to Aβ deposits using an inverted Zeiss (Axio Observer.Z1) LSM 780 microscope equipped with a 32 channel QUASAR GaAsP spectral array detector (Carl Zeiss, Oberkochen, Germany) or a Leica DM6000 B

fluorescence microscope (Leica, Germany) equipped with a SpectraCube module (Applied Spectral Imaging, Israel). For excitation of the D-A-D ligands at 405 nm and 535 nm, a Laser diode 405nm CW/PS and a pulsed tunable In Tune laser were utilized, whereas an Argon laser was used for excitation of the heptameric oligothiophenes at 458 nm. Spectra were collected from 10 individual Aβ deposits (Aβ core plaques or CAA) for each ligand.

Immunohistochemistry (D-A-D heptameric ligand and antibody double labeling)

Frozen brain sections (10 or 20 µm thickness) from transgenic APP23 mice were fixed in 70% EtOH for 3 min at 4°C, rehydrated in de-ionized water (2x2 min) and then incubated in PBS for 10 min at RT. After 1 h incubation in PBS with 0.1% triton x-100 (PBS-T) and 5% normal goat serum (blocking buffer), the 4G8 or the 6E10 anti-amyloid-β antibody (Biolegend, San Diego, CA, USA) was diluted 1:500 in blocking buffer and added to the sections. After 16 h at 4°C, unbound antibody was removed by washing with PBS-T (3x10 min). The sections were then incubated for 1 h at RT with goat anti-mouse secondary antibody conjugated to Alexa Fluor 594 (Thermo Fisher Scientific, Waltham, MA, USA), diluted 1:400 in blocking buffer. After washing with PBS (3x10 min), the sections were incubated with 600 nM LL-1 or LL-3 ligand diluted in PBS for 30 min at RT. The sections were then rinsed in PBS and mounted with Dako mounting medium for fluorescence (Dako Cytomation, Glostrup, Denmark), which was allowed to solidify before analyzing the result using an inverted Zeiss (Axio Observer.Z1) LSM 780 microscope (Carl Zeiss, Oberkochen, Germany). The following excitation/emission wavelengths were used to visualize the antibody and the ligand staining: 595/600-640 (Alexa Fluor 594) and 535/680-760 (LL-1 and LL-3).

References

[1] H. Shirani, M. Linares, C. Sigurdson, M. Lindgren, P. Norman, K. P. R. Nilsson, *Chemistry* **2015**, *21*, 15133-15137.

[2] R. A. Simon, H. Shirani, K. O. A. Åslund, M. Bäck, V. Haroutunian, S. Gandy, K. P. R. Nilsson, *Chemistry* **2014**, *20*, 12537–12543.

[3] T. Klingstedt, A. Åslund, R. A. Simon, L. B. G. Johansson, J. J. Mason, S. Nyström, P. Hammarström, K. P. R. Nilsson, *Org. Biomol. Chem.* **2011**, *9*, 8356–8370.

[4] T. Klingstedt, H. Shirani, J. Mahler, B. M. Wegenast-Braun, S. Nyström, M. Goedert, M. Jucker, K. P. R. Nilsson, *Chemistry.* **2015**, *21*, 9072–9082.

[5] A. Åslund, C. Sigurdson, T. Klingstedt, S. Grathwohl, T. Bolmont, D. L. Dickstein, E. Glimsdal, S. Prokop, M. Lindgren, P. Konradsson, D. M. Holtzman, P. R. Hof, F. L.

Heppner, S. Gandy, M. Jucker, A. Aguzzi, P. Hammarström, K. P. R. Nilsson, *ACS Chem. Biol.* **2009**, *4*, 673–684.

[6] A. Åslund, A. Herland, P. Hammarström, K. P. R. Nilsson, B.-H. Jonsson, O. Inganäs, P. Konradsson, *Bioconjugate Chem*. **2007**, *18*, 1860–1868.

[7] A. Åslund, K. P. R. Nilsson, P. Konradsson, *Synthesis of a pentathiophene fluorescent probe, 4',3''''-bis-carboxymethyl-[2,2';5',2'';5'',2''';5''',2'''']quinquethiophene-5,5'*, **2010**, DOI:10.1038/nprot.2010.24

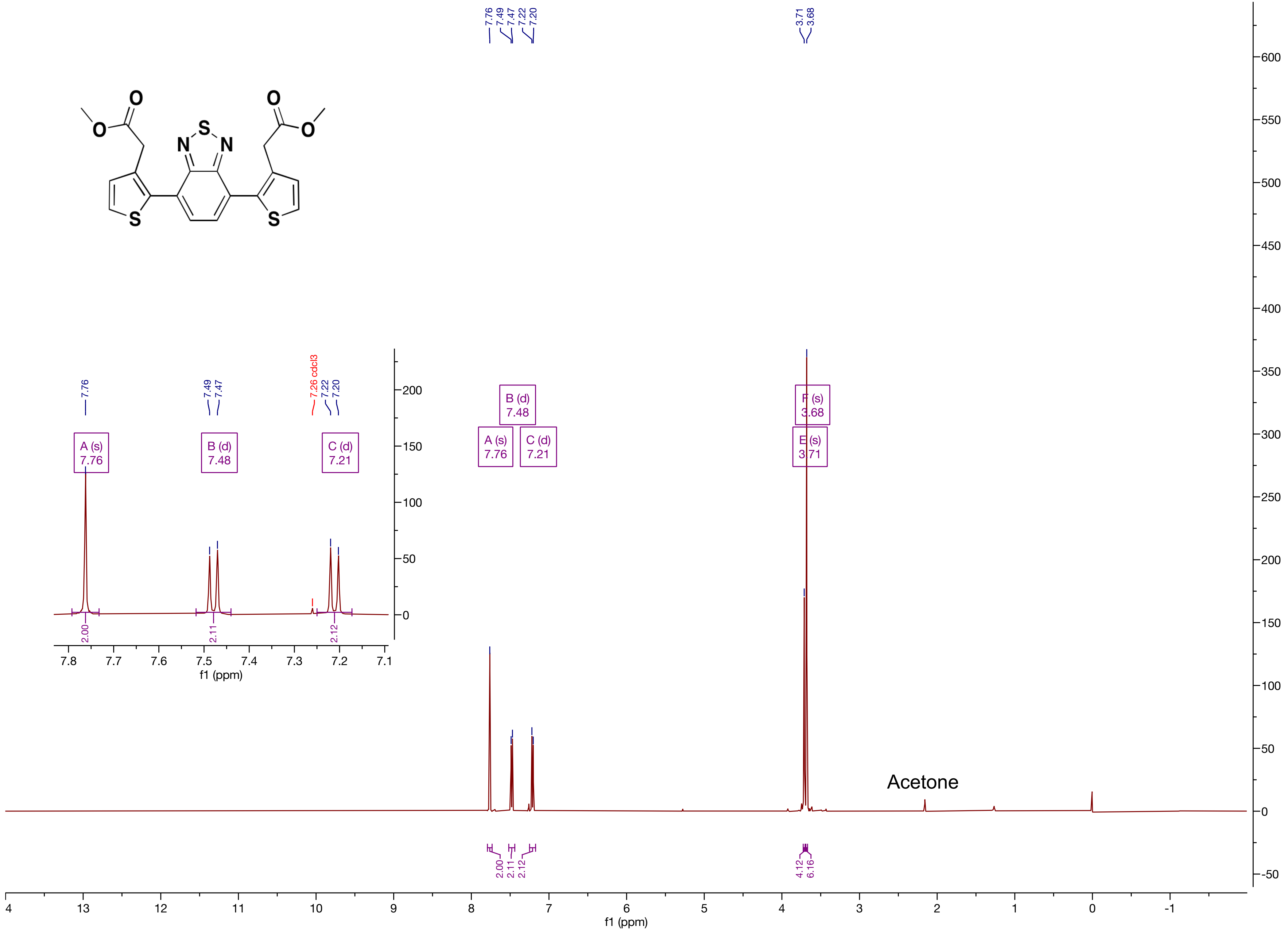

7.76
7.49
7.47
7.22
7.20
3.71
3.68
7.26 cdcl3
A (s) 7.76
B (d) 7.48
C (d) 7.21
E (s) 3.71
F (s) 3.68
2.00
2.11
2.12
4.12
6.16
Acetone
f1 (ppm)

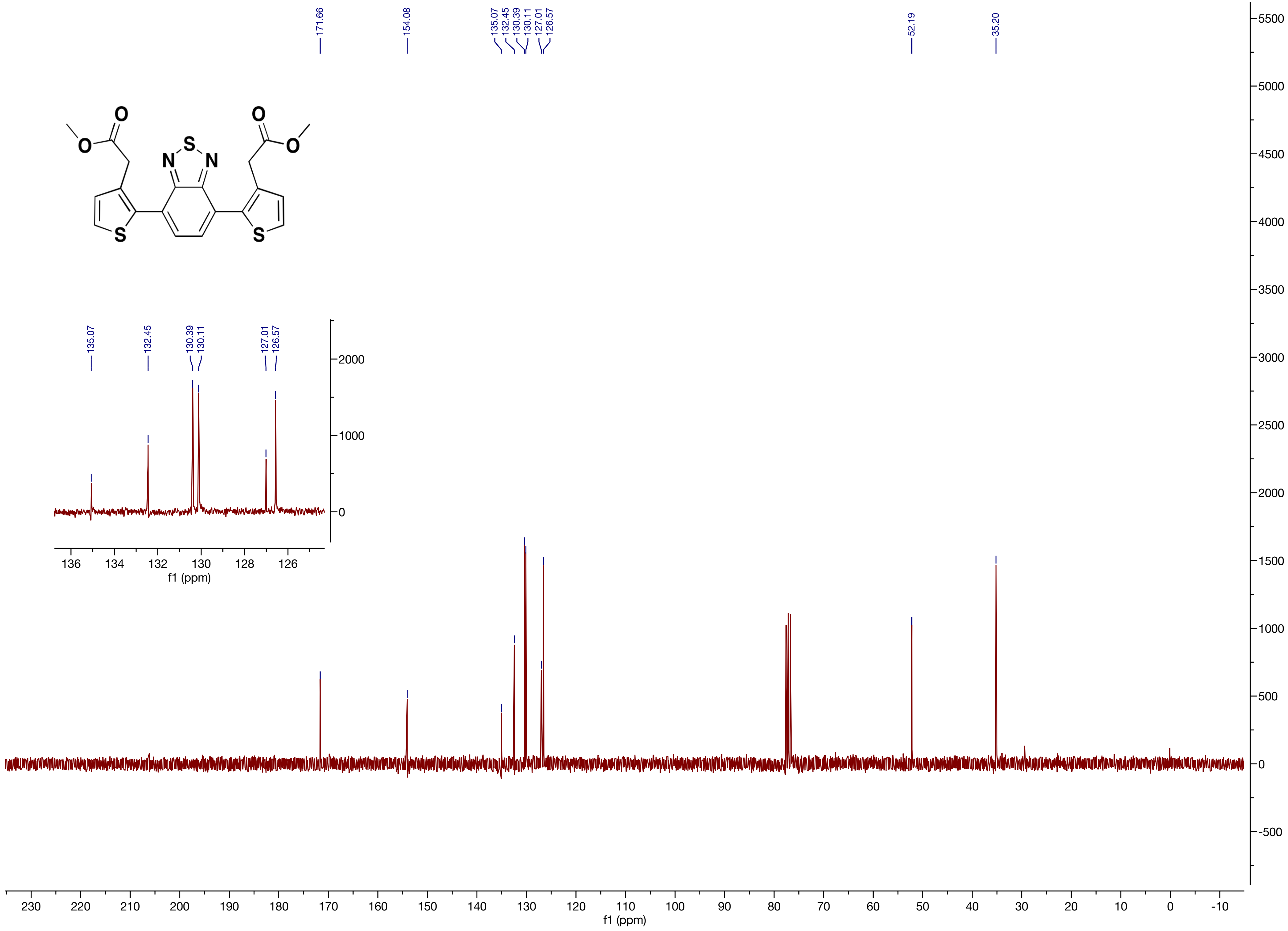

171.66
154.08
135.07
132.45
130.39
130.11
127.01
126.57
52.19
35.20
O
O
S
N
N
S
S
f1 (ppm)

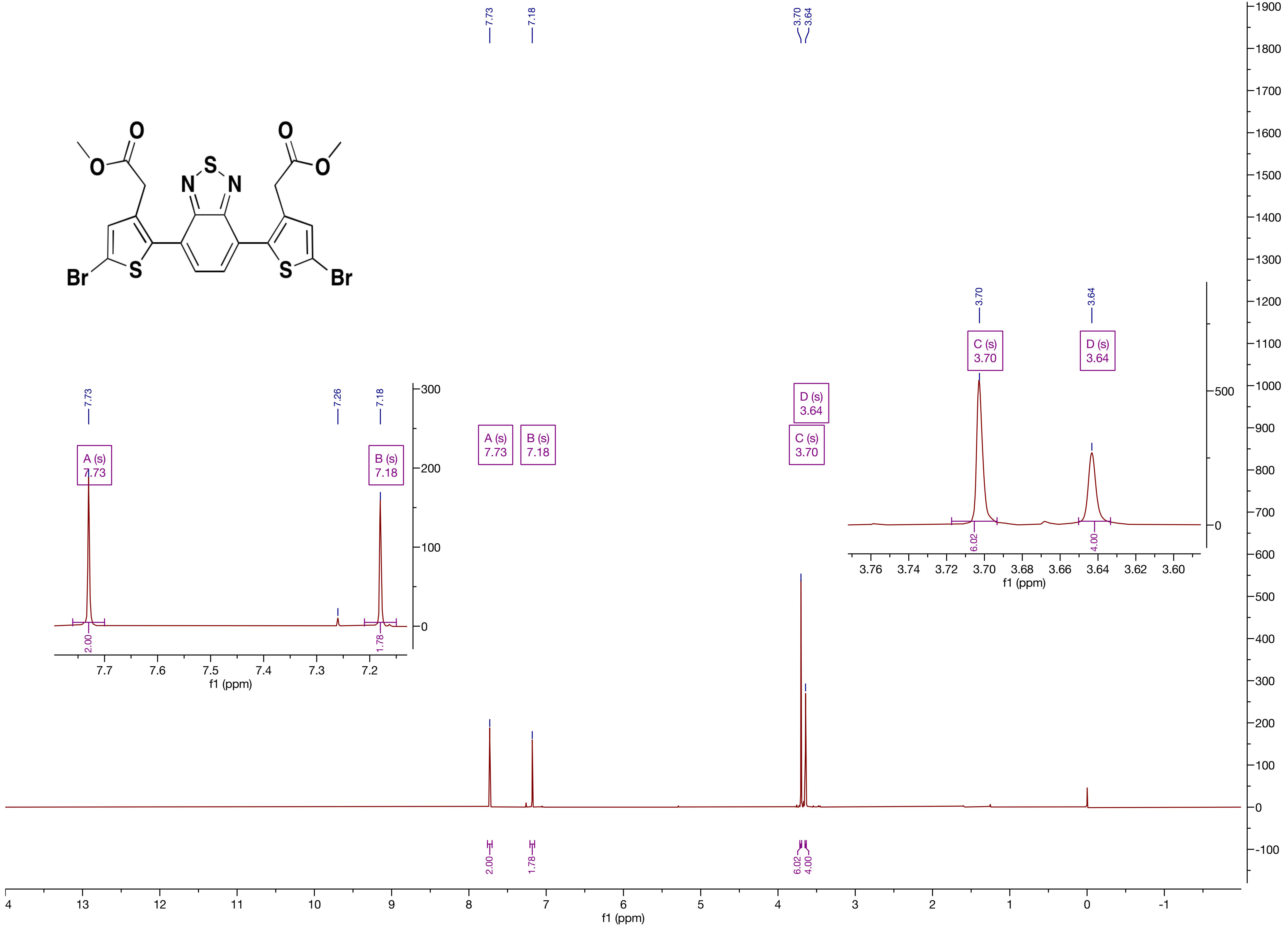

7.73
7.18
3.70
3.64
A (s) 7.73
B (s) 7.18
C (s) 3.70
D (s) 3.64
7.26
2.00
1.78
6.02
4.00
Br
S
N
O
f1 (ppm)

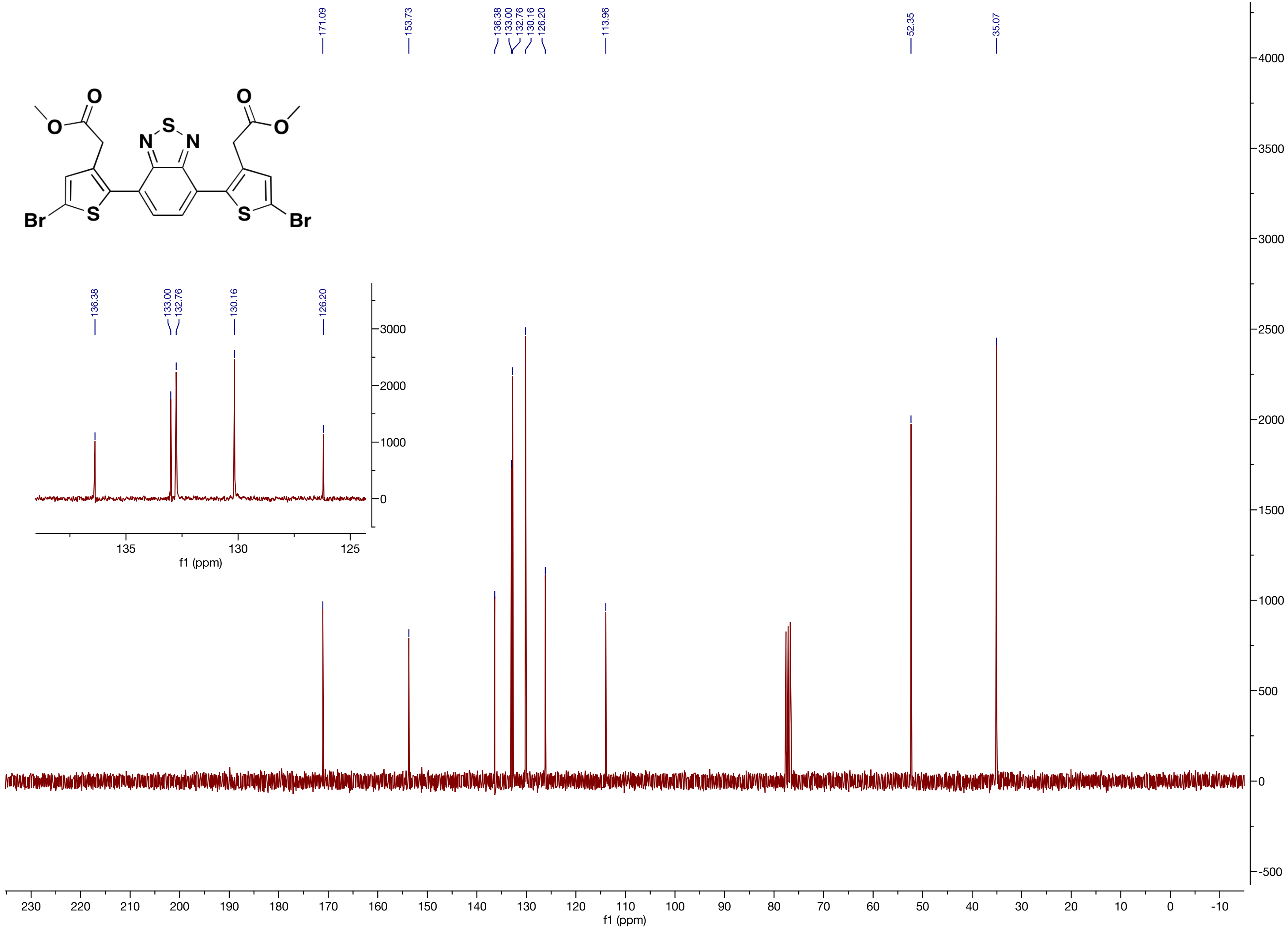

171.09
153.73
136.38
133.00
132.76
130.16
126.20
113.96
52.35
35.07
O
O
N
S
N
O
O
Br
S
S
Br
f1 (ppm)
f1 (ppm)

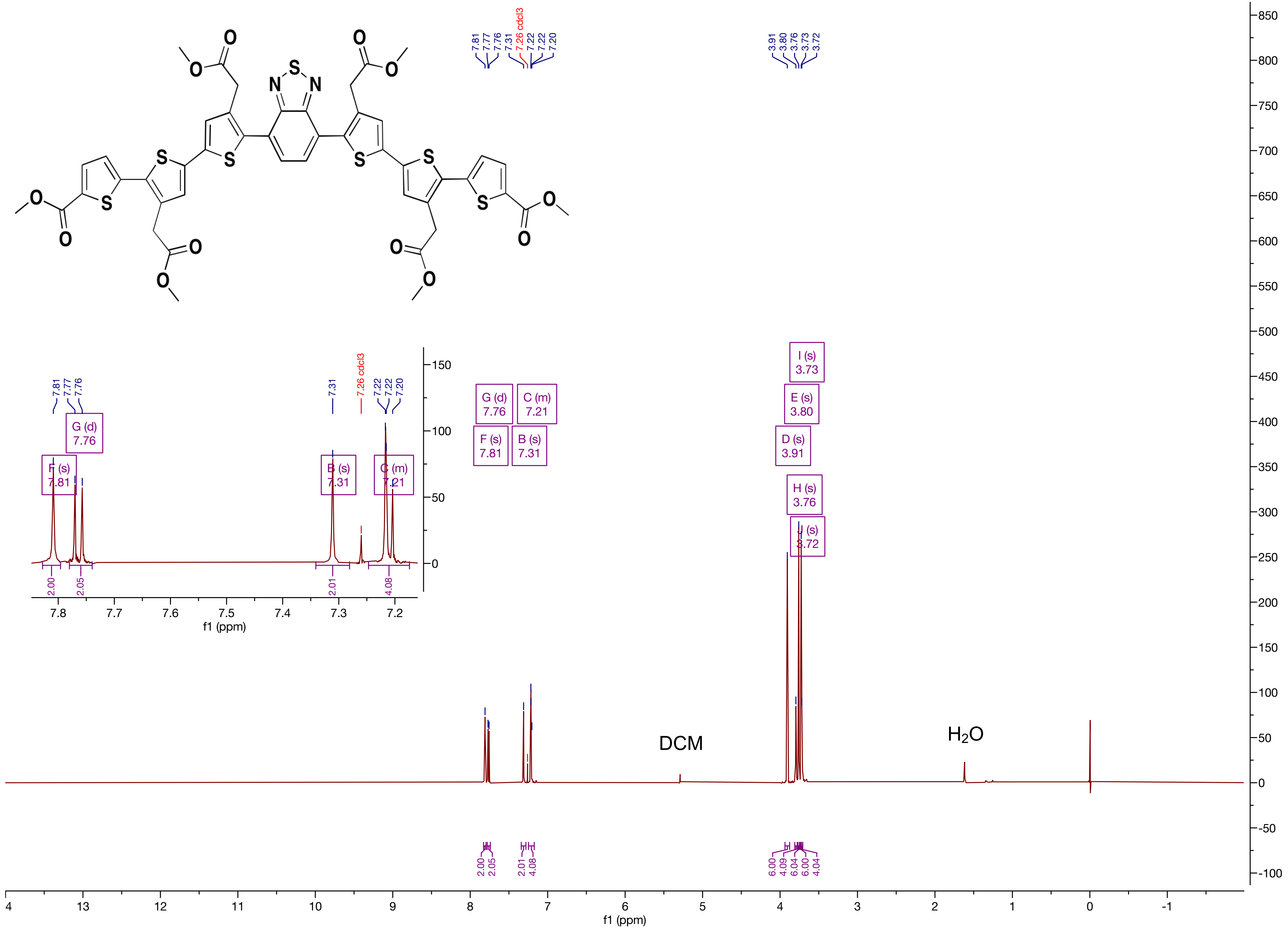
7.81
7.77
7.76
7.31
7.26 cdcl3
7.22
7.22
7.20
3.91
3.80
3.76
3.73
3.72
G (d) 7.76
C (m) 7.21
F (s) 7.81
B (s) 7.31
I (s) 3.73
E (s) 3.80
D (s) 3.91
H (s) 3.76
J (s) 3.72
2.00
2.05
2.01
4.08
6.00
4.09
6.04
6.00
4.04
DCM
H_2O
f1 (ppm)

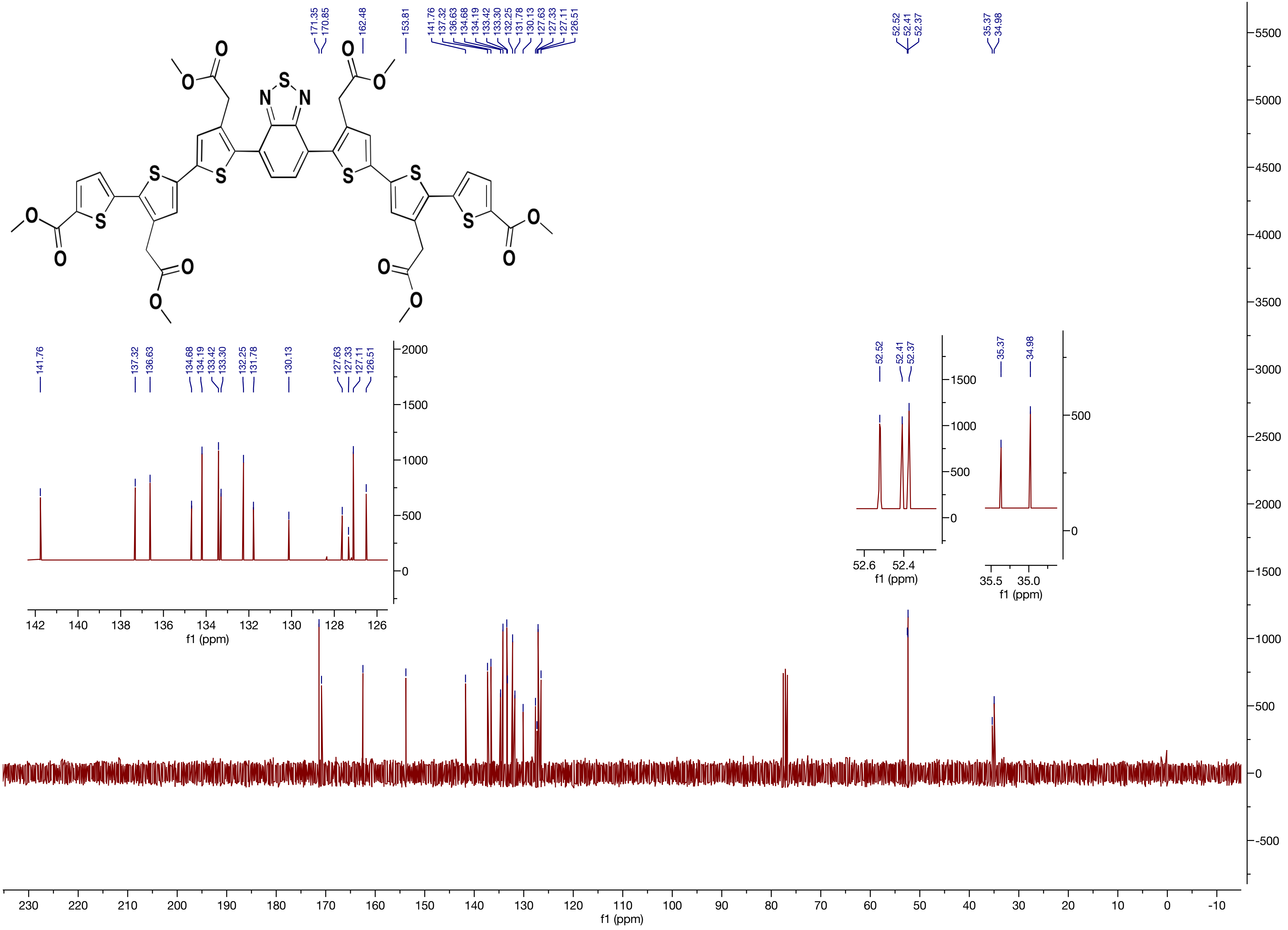

171.35
170.85
162.48
153.81
141.76
137.32
136.63
134.68
134.19
133.42
133.30
132.25
131.78
130.13
127.63
127.33
127.11
126.51
52.52
52.41
52.37
35.37
34.98
f1 (ppm)

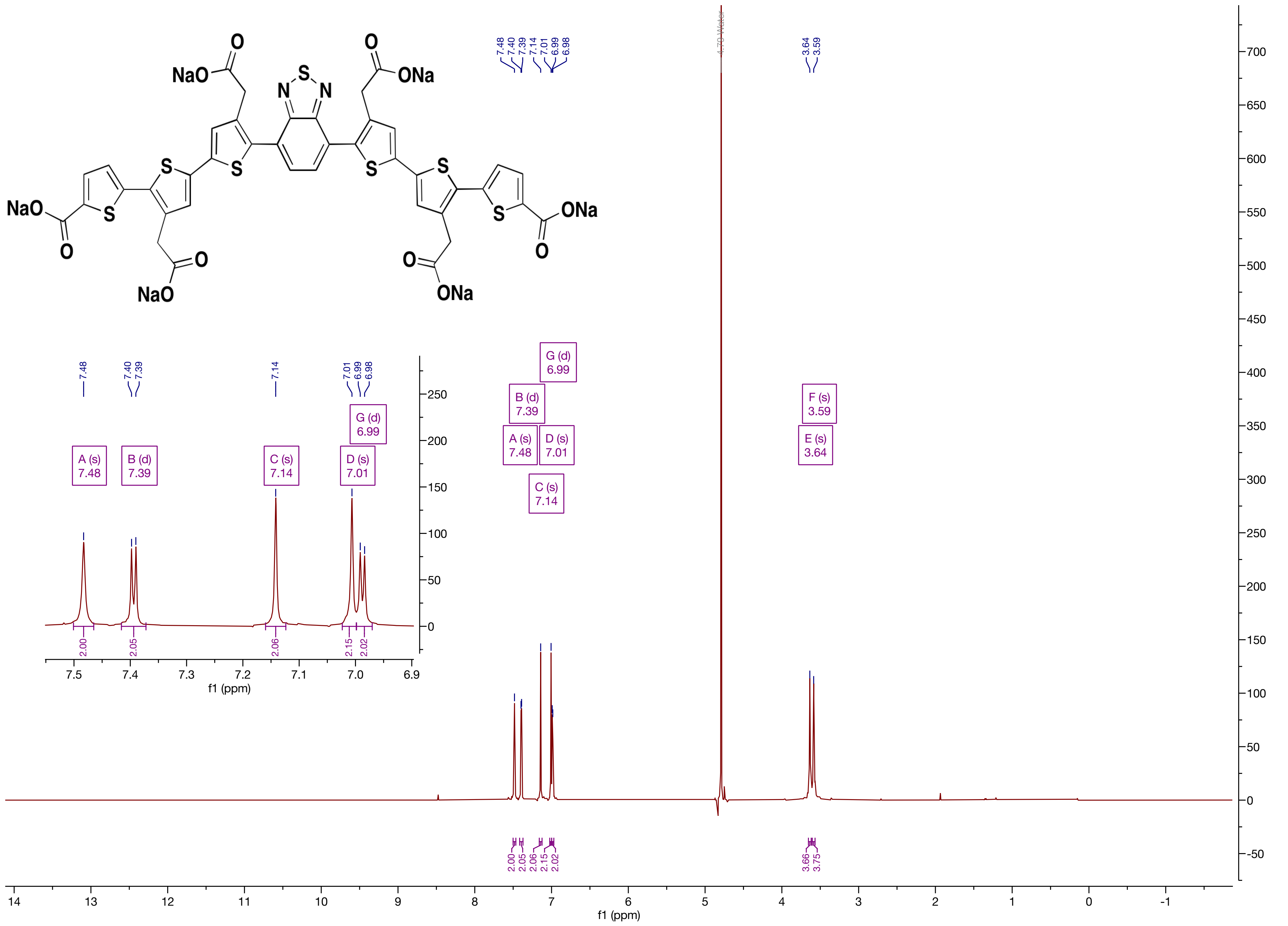
NaO
O
NaO
O
S
S
S
N
S
N
O
ONa
S
S
S
ONa
O
O
NaO
O
ONa
7.48
7.40
7.39
7.14
7.01
6.99
6.98
4.79 Water
3.64
3.59
G (d) 6.99
B (d) 7.39
A (s) 7.48
D (s) 7.01
C (s) 7.14
F (s) 3.59
E (s) 3.64
2.00
2.05
2.06
2.15
2.02
3.66
3.75
f1 (ppm)

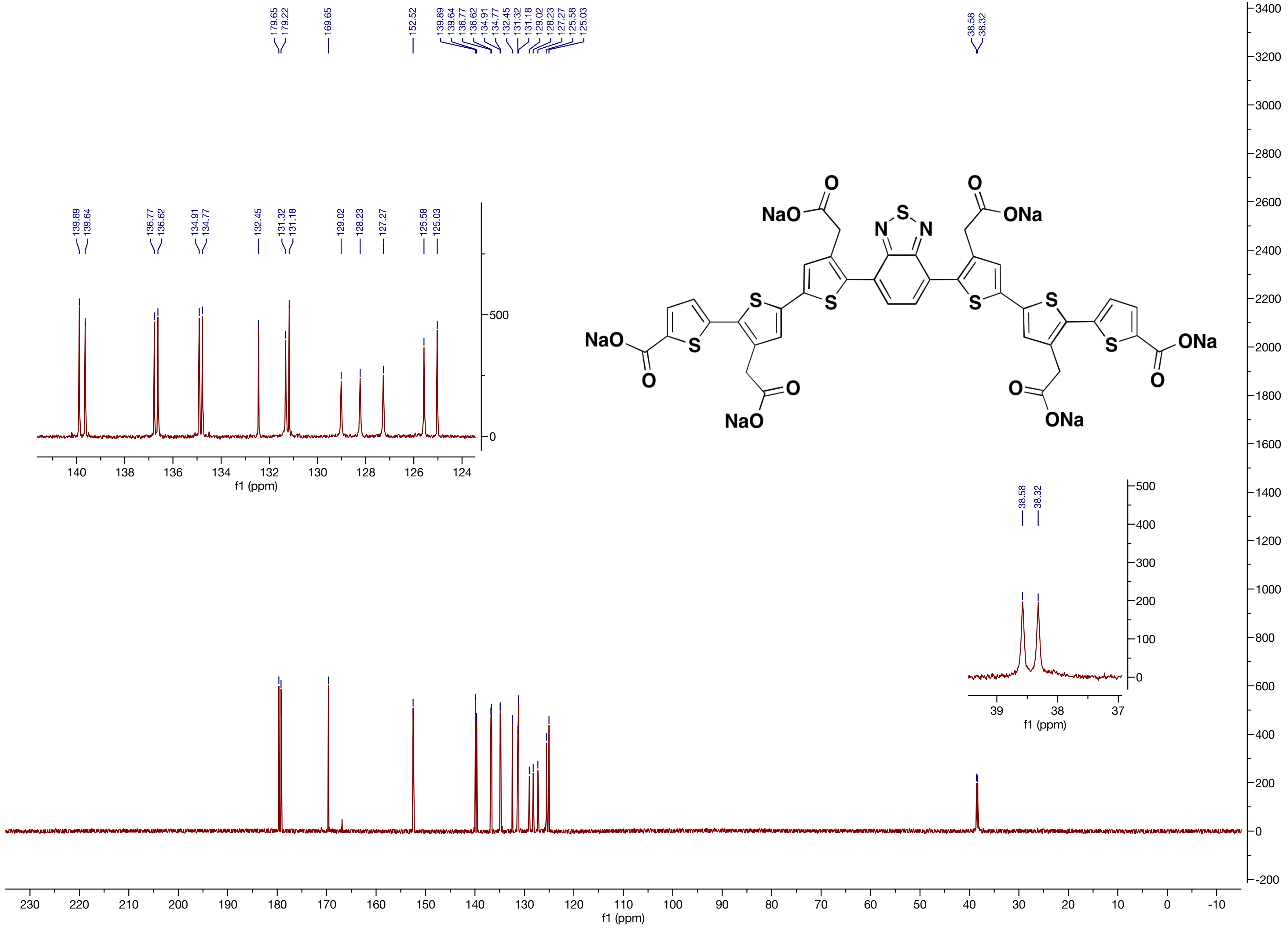

179.65
179.22
169.65
152.52
139.89
139.64
136.77
136.62
134.91
134.77
132.45
131.32
131.18
129.02
128.23
127.27
125.58
125.03
38.58
38.32
NaO
O
S
N
N
ONa
NaO
S
S
S
S
S
ONa
O
O
O
NaO
ONa
O
140
138
136
134
132
130
128
126
124
f1 (ppm)
500
0
500
400
300
200
100
0
39
38
37
f1 (ppm)
3400
3200
3000
2800
2600
2400
2200
2000
1800
1600
1400
1200
1000
800
600
400
200
0
-200
230
220
210
200
190
180
170
160
150
140
130
120
110
100
90
80
70
60
50
40
30
20
10
0
-10
f1 (ppm)

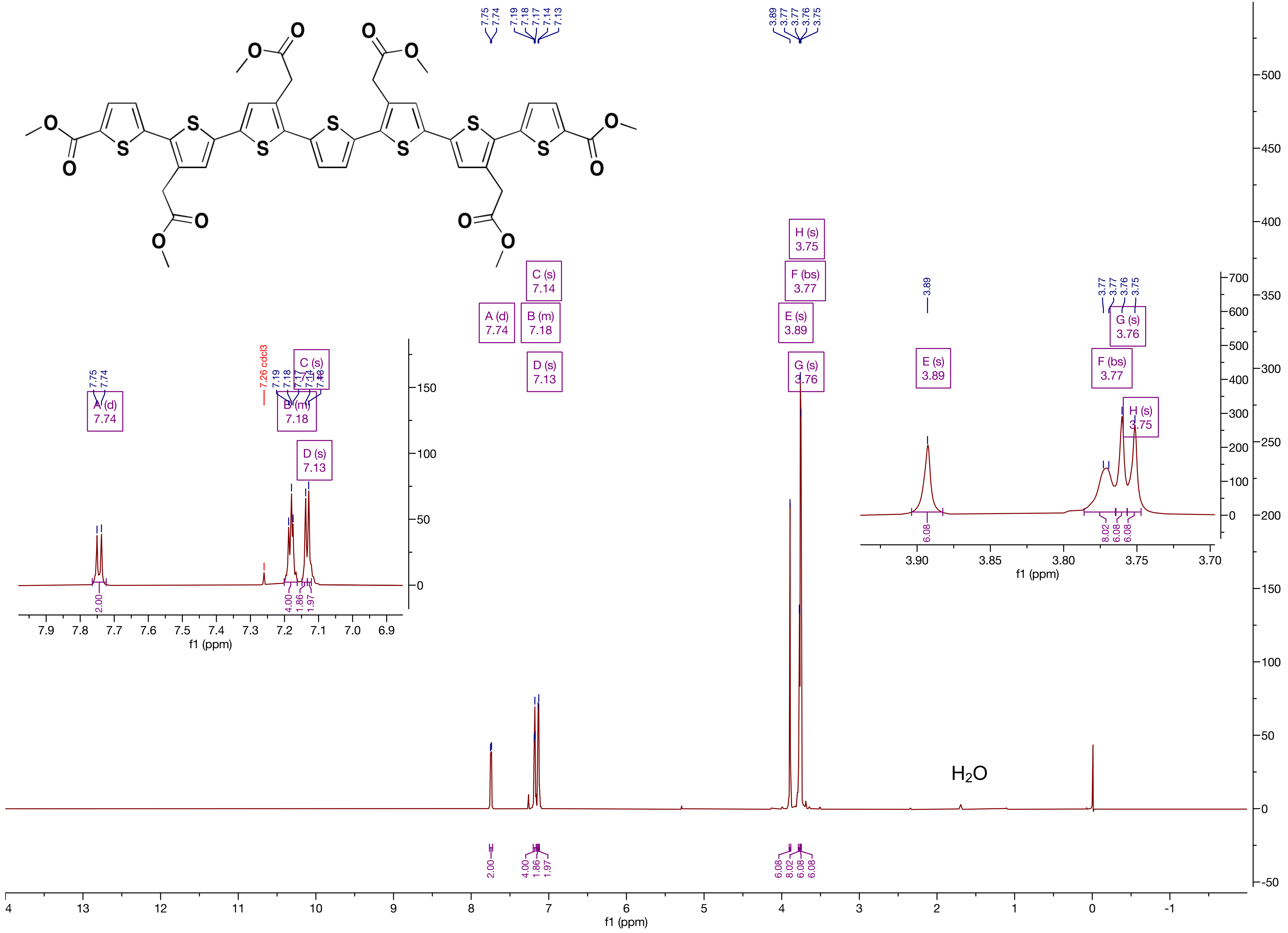

7.75
7.74
7.19
7.18
7.17
7.14
7.13
3.89
3.77
3.77
3.76
3.75
A (d) 7.74
B (m) 7.18
C (s) 7.14
D (s) 7.13
E (s) 3.89
F (bs) 3.77
G (s) 3.76
H (s) 3.75
7.26 cdcl3
2.00
4.00
1.86
1.97
6.08
8.02
6.08
6.08
H_2O
f1 (ppm)

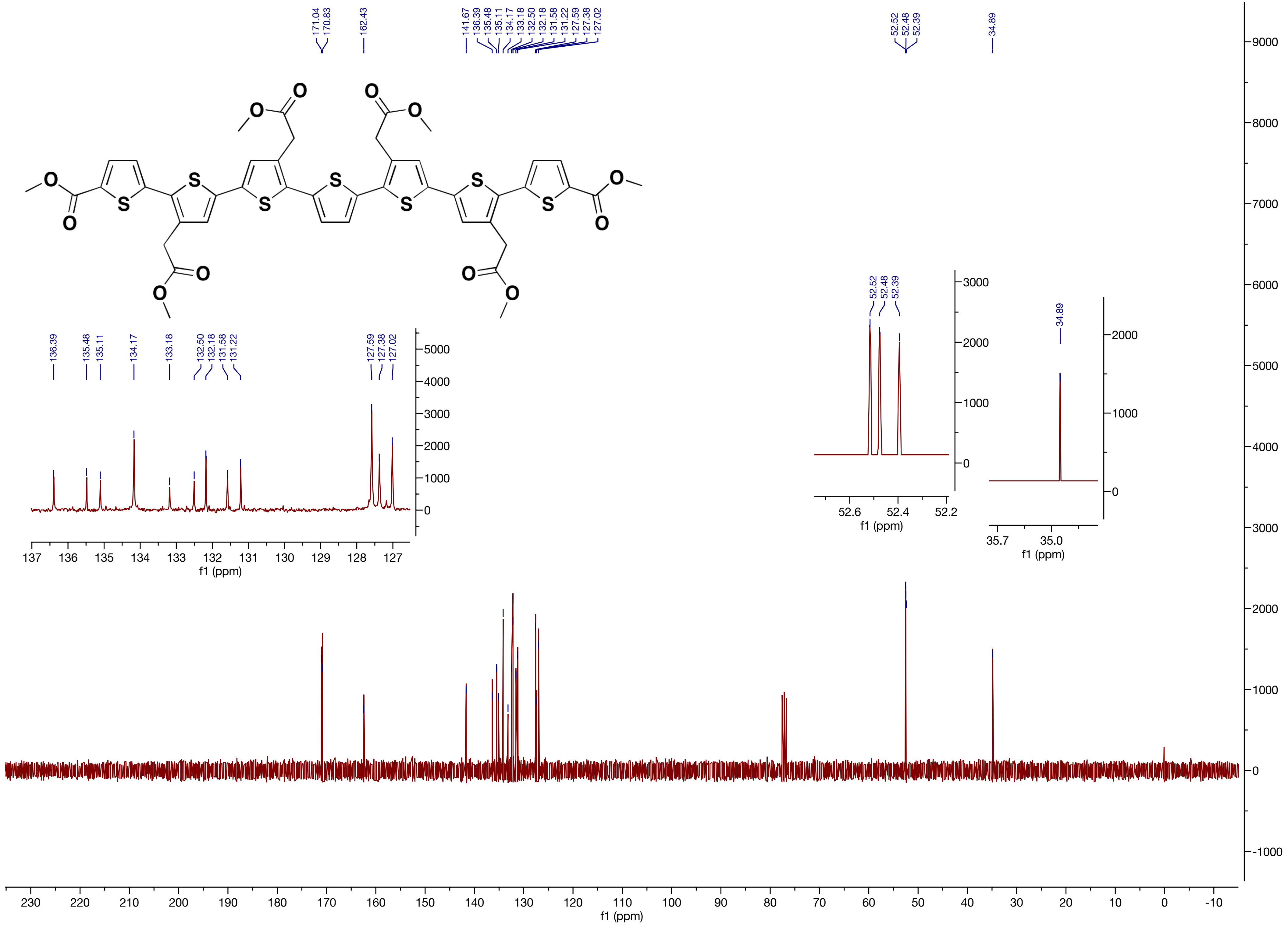

171.04
170.83
162.43
141.67
136.39
135.48
135.11
134.17
133.18
132.50
132.18
131.58
131.22
127.59
127.38
127.02
52.52
52.48
52.39
34.89
f1 (ppm)

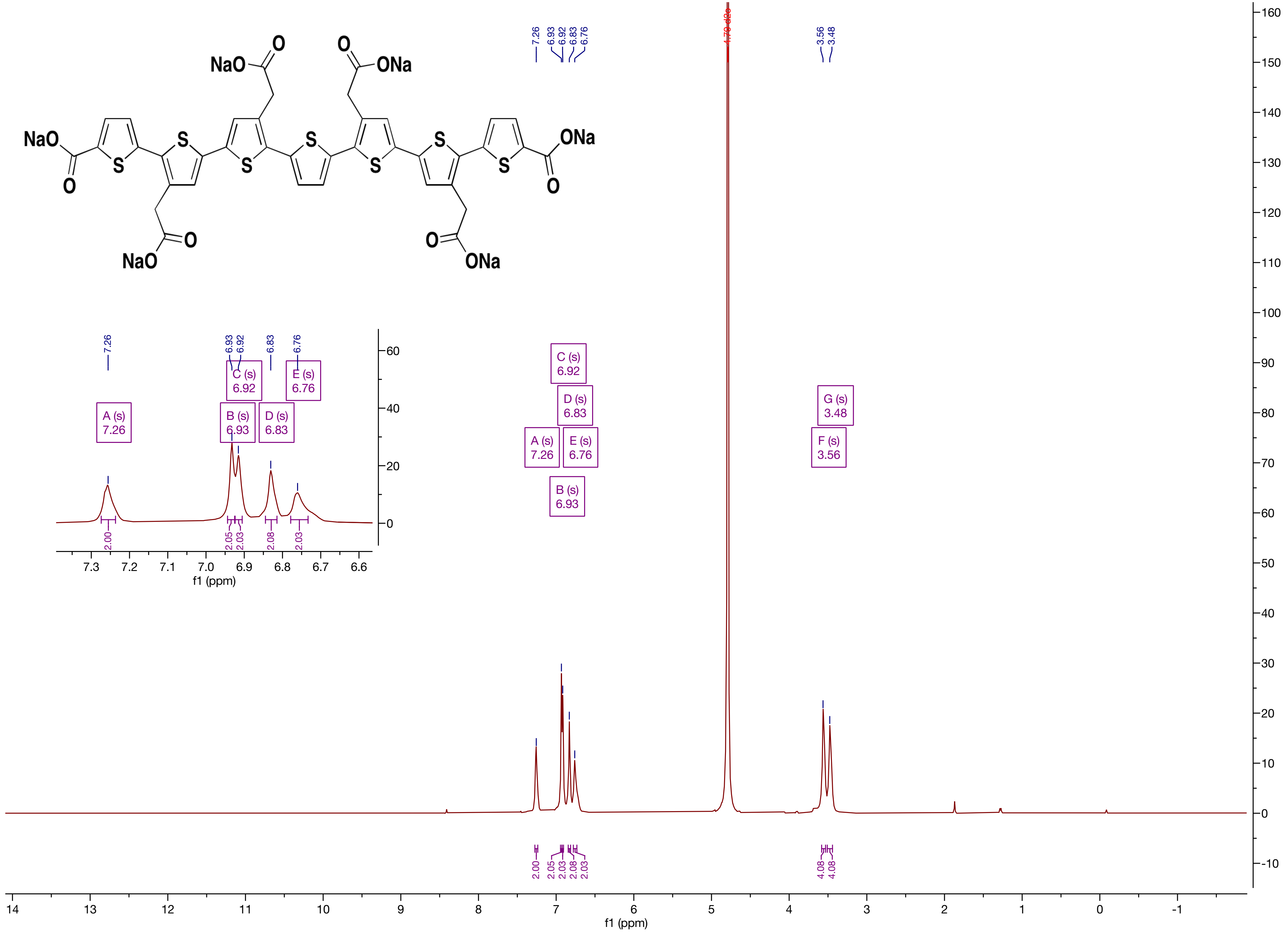

NaO
O
ONa
NaO
S
ONa
O
NaO
ONa
7.26
6.93
6.92
6.83
6.76
4.79 d2o
3.56
3.48
A (s)
7.26
B (s)
6.93
C (s)
6.92
D (s)
6.83
E (s)
6.76
F (s)
3.56
G (s)
3.48
2.00
2.05
2.03
2.08
2.03
4.08
4.08
f1 (ppm)

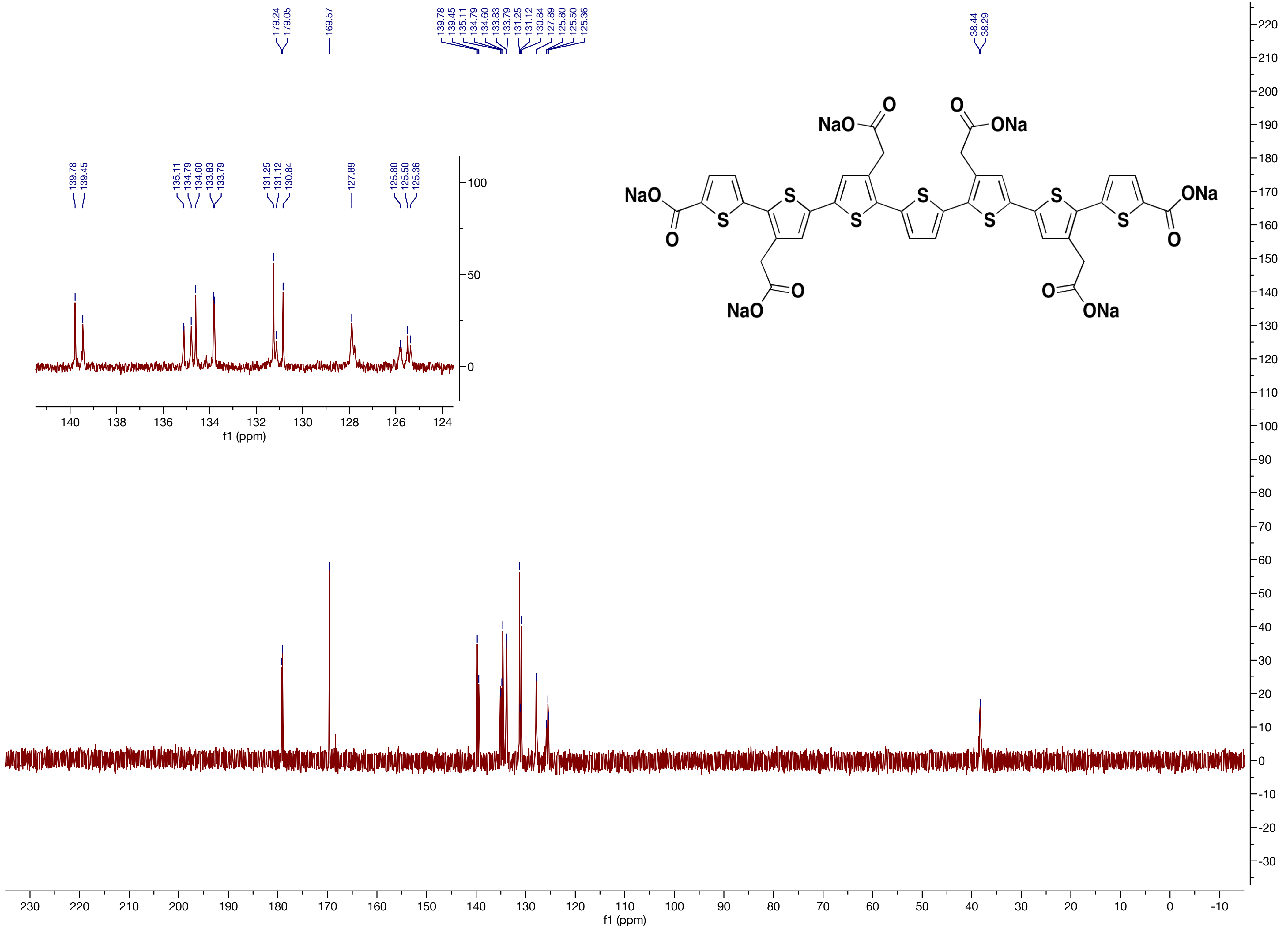

179.24
179.05
169.57
139.78
139.45
135.11
134.79
134.60
133.83
133.79
131.25
131.12
130.84
127.89
125.80
125.50
125.36
38.44
38.29
NaO
ONa
S
O
f1 (ppm)

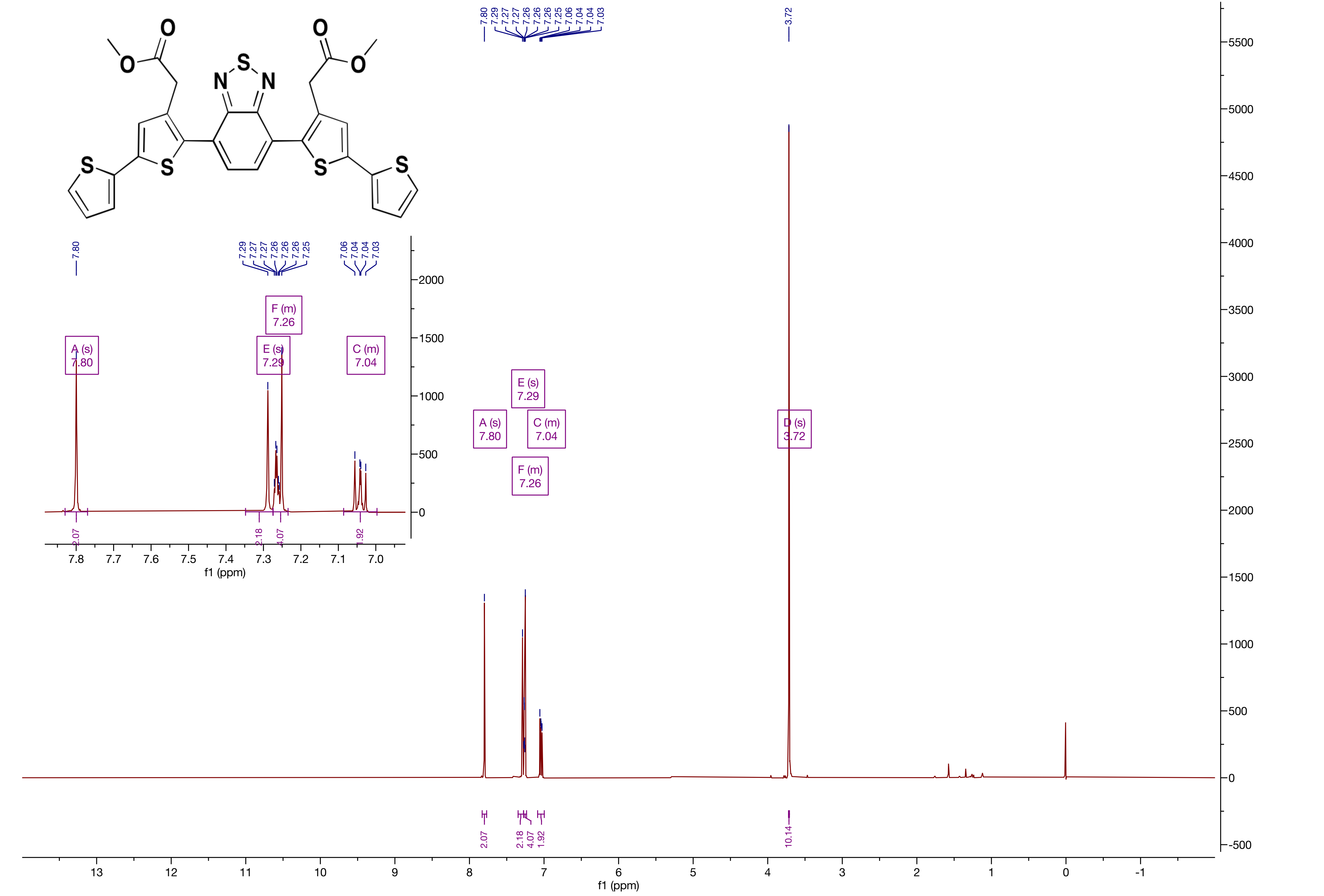

O
O
S
N
N
S
S
O
O
S
S
7.80
7.29
7.27
7.27
7.26
7.26
7.26
7.25
7.06
7.04
7.04
7.03
3.72
A (s)
7.80
E (s)
7.29
C (m)
7.04
F (m)
7.26
D (s)
3.72
2.07
2.18
4.07
1.92
10.14
f1 (ppm)

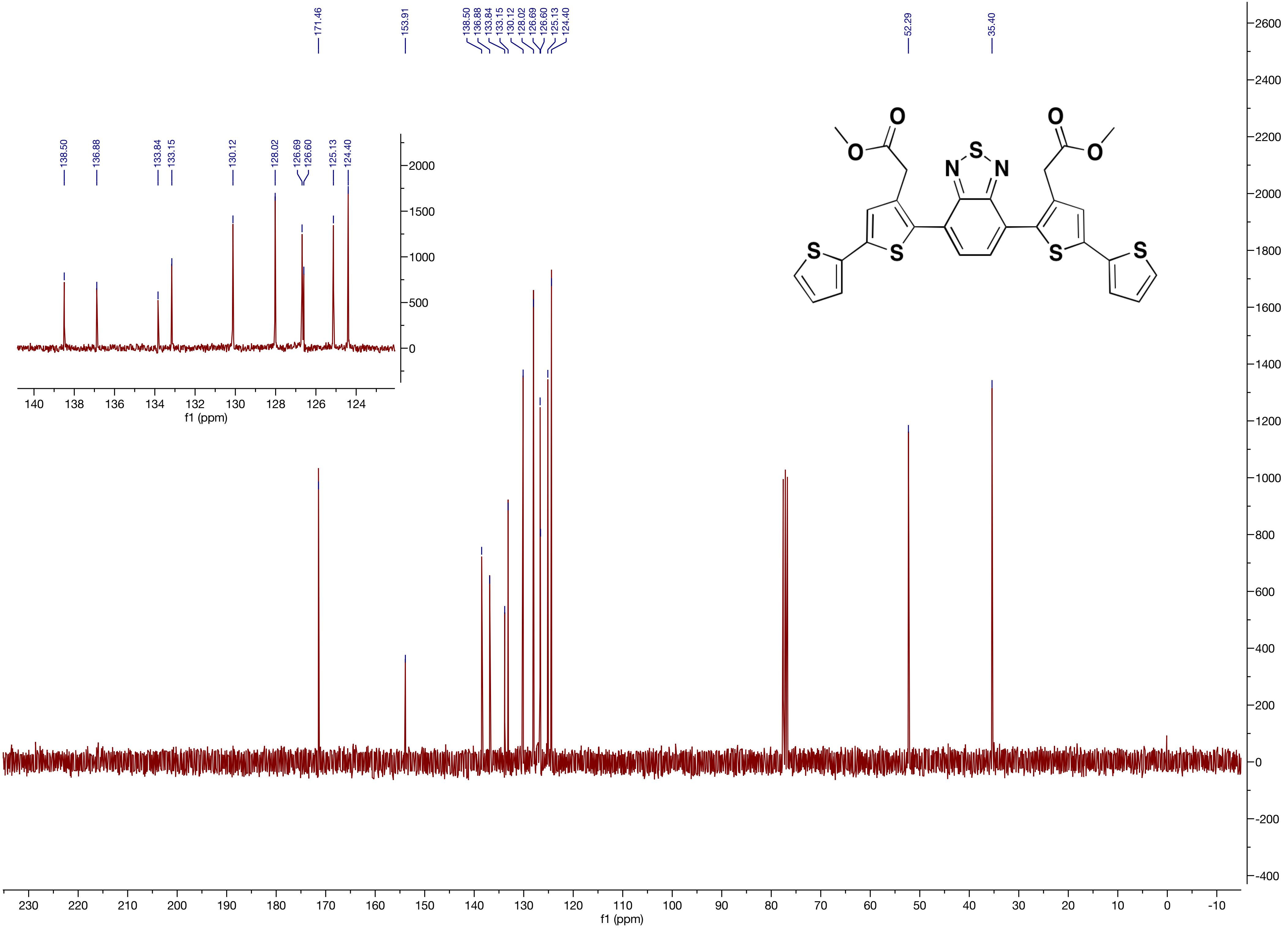

171.46
153.91
138.50
136.88
133.84
133.15
130.12
128.02
126.69
126.60
125.13
124.40
52.29
35.40
f1 (ppm)
f1 (ppm)

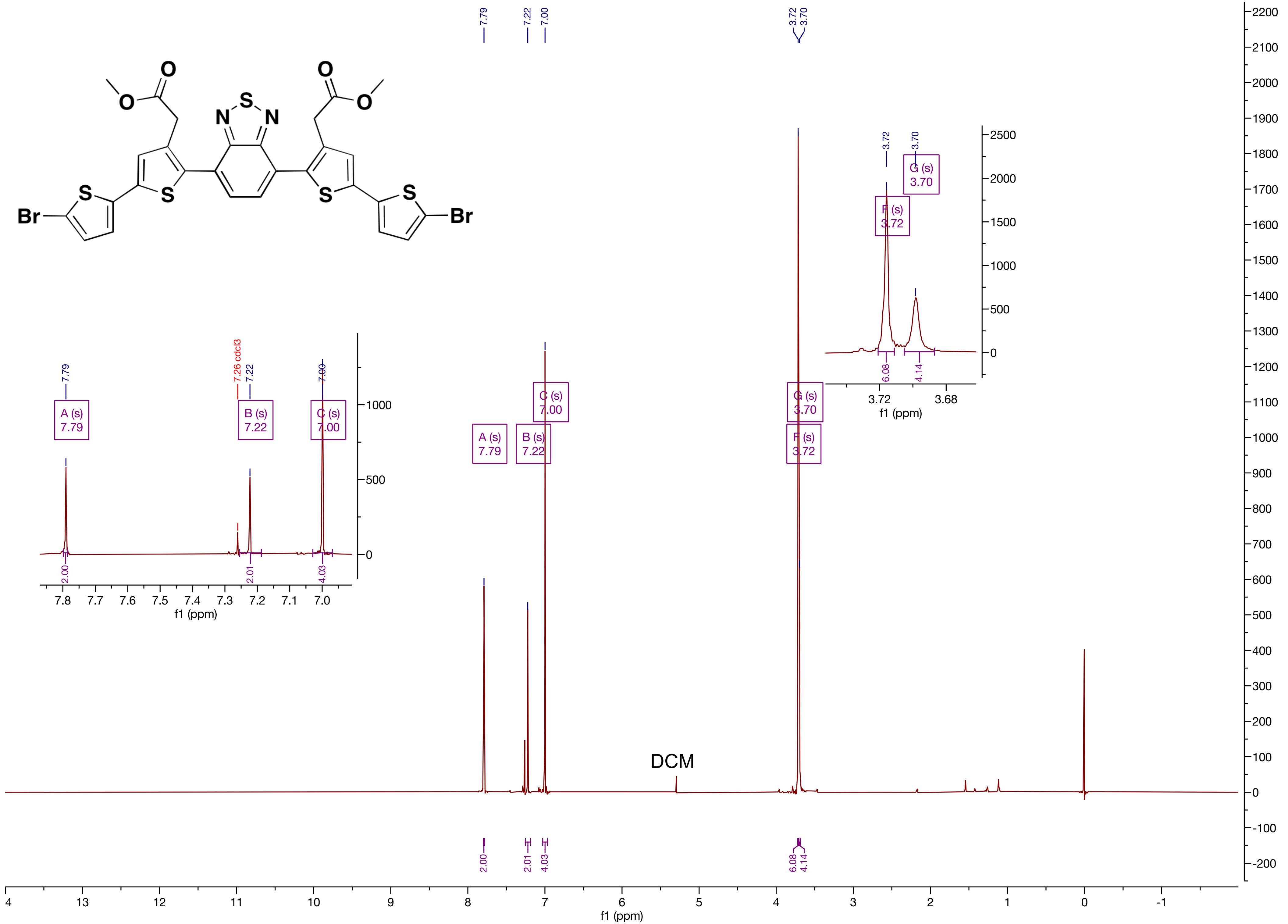

7.79
7.22
7.00
3.72
3.70
O
O
N
S
N
S
S
S
S
Br
Br
7.26 cdcl3
A (s)
7.79
B (s)
7.22
C (s)
7.00
F (s)
3.72
G (s)
3.70
2.00
2.01
4.03
6.08
4.14
DCM
f1 (ppm)

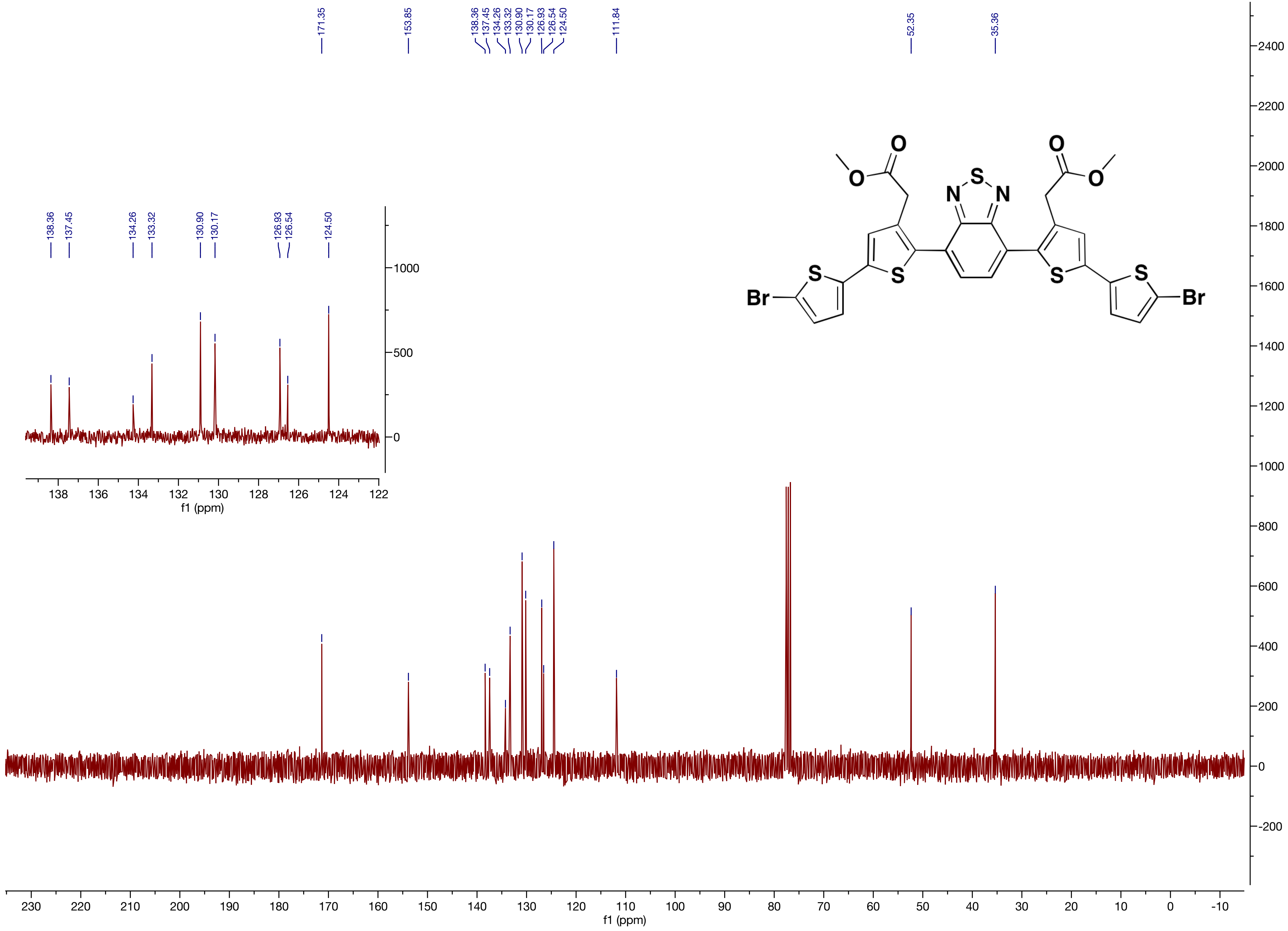

171.35
153.85
138.36
137.45
134.26
133.32
130.90
130.17
126.93
126.54
124.50
111.84
52.35
35.36
f1 (ppm)
Br
S
S
N
S
N
O
O
O
O
S
S
Br

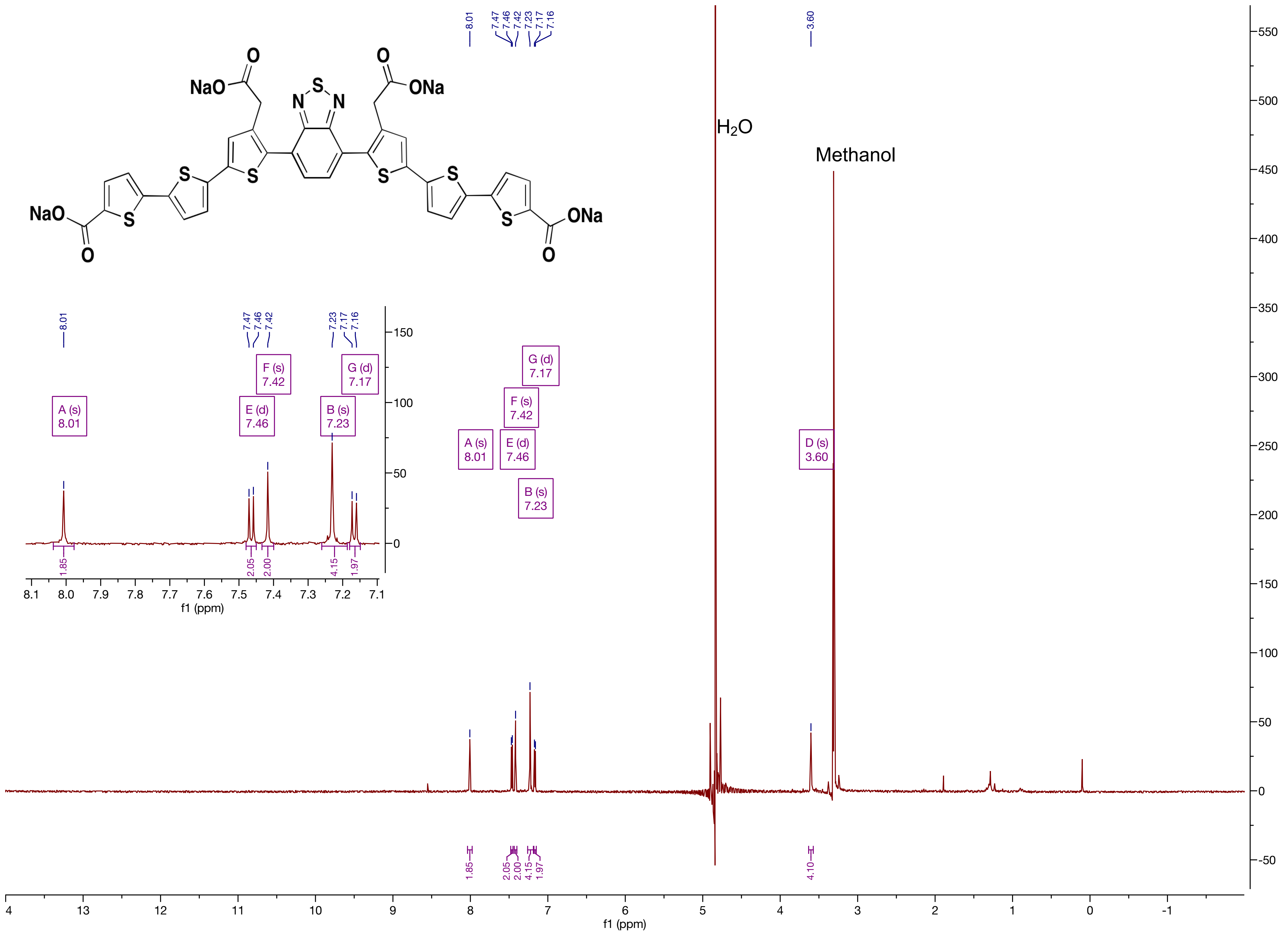

NaO
O
S
N
N
ONa
O
S
S
S
S
S
NaO
O
ONa
O
8.01
7.47
7.46
7.42
7.23
7.17
7.16
3.60
H2O
Methanol
A (s)
8.01
E (d)
7.46
F (s)
7.42
B (s)
7.23
G (d)
7.17
D (s)
3.60
1.85
2.05
2.00
4.15
1.97
4.10
f1 (ppm)

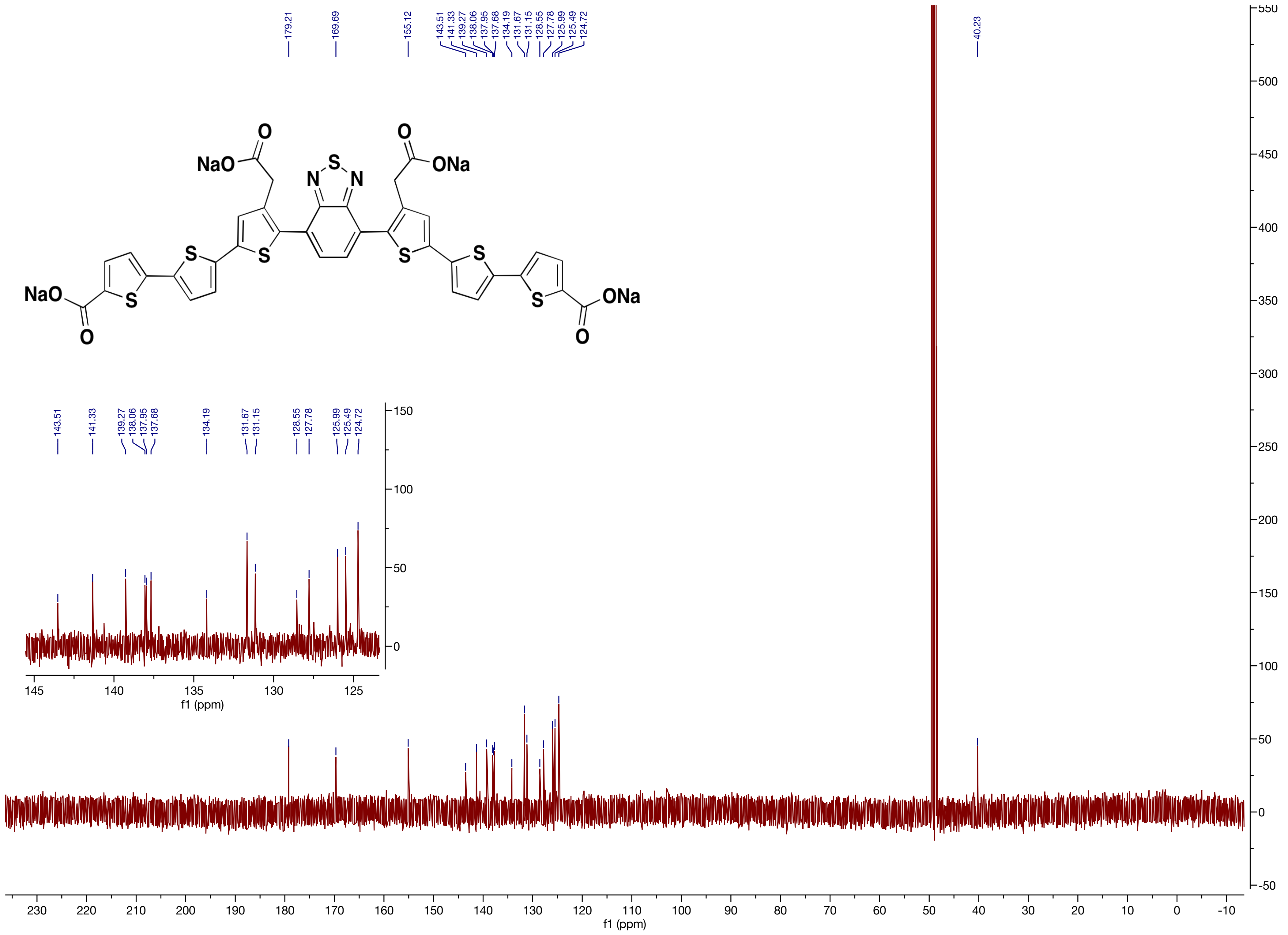

179.21
169.69
155.12
143.51
141.33
139.27
138.06
137.95
137.68
134.19
131.67
131.15
128.55
127.78
125.99
125.49
124.72
40.23
NaO
O
S
N
N
ONa
f1 (ppm)

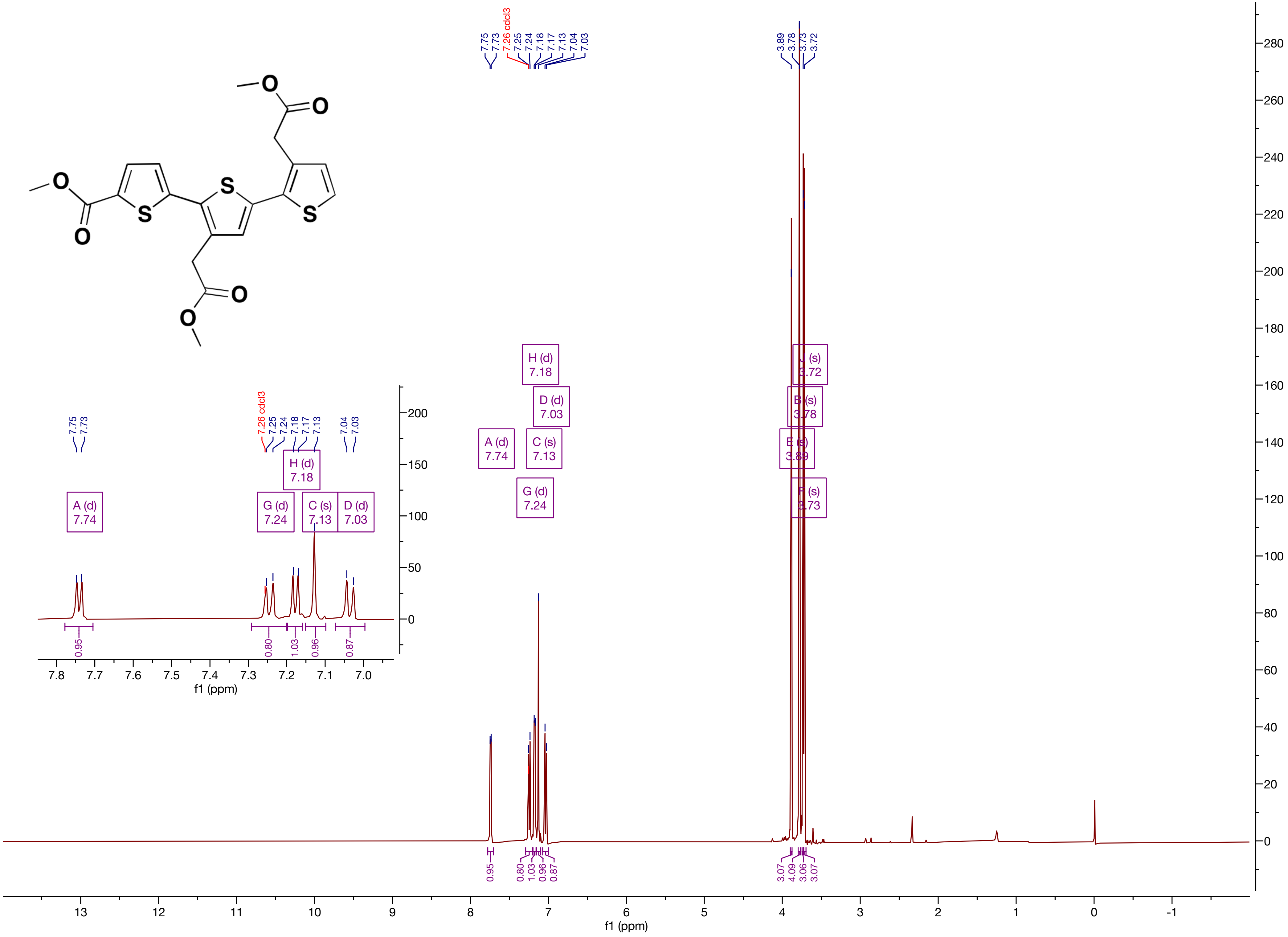

7.75
7.73
7.26 cdcl3
7.25
7.24
7.18
7.17
7.13
7.04
7.03
3.89
3.78
3.73
3.72
A (d) 7.74
G (d) 7.24
H (d) 7.18
C (s) 7.13
D (d) 7.03
E (s) 3.89
B (s) 3.78
F (s) 3.73
J (s) 3.72
0.95
0.80
1.03
0.96
0.87
3.07
4.09
3.06
3.07
f1 (ppm)

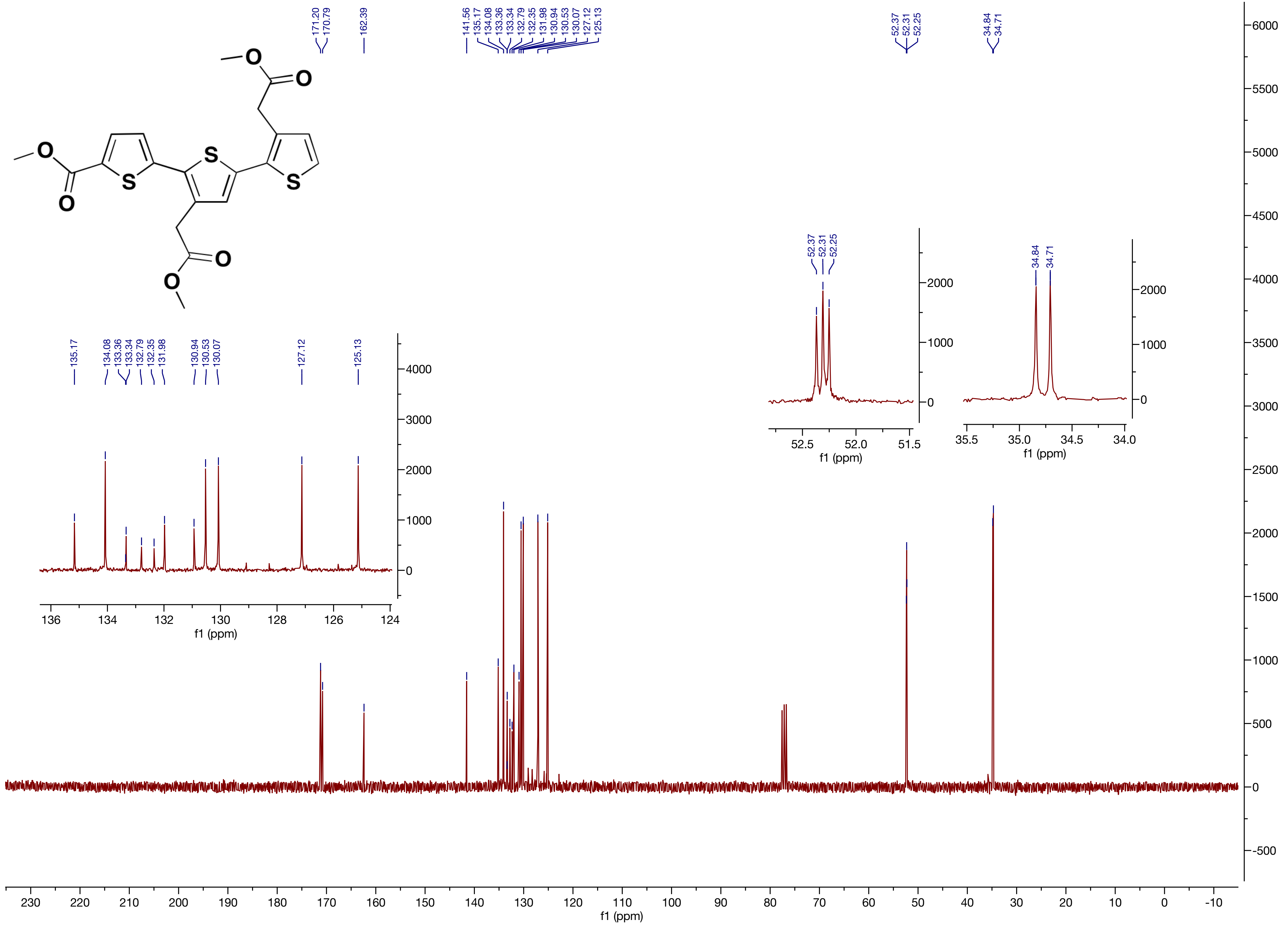
171.20
170.79
162.39
141.56
135.17
134.08
133.36
133.34
132.79
132.35
131.98
130.94
130.53
130.07
127.12
125.13
52.37
52.31
52.25
34.84
34.71
O
O
S
S
S
f1 (ppm)

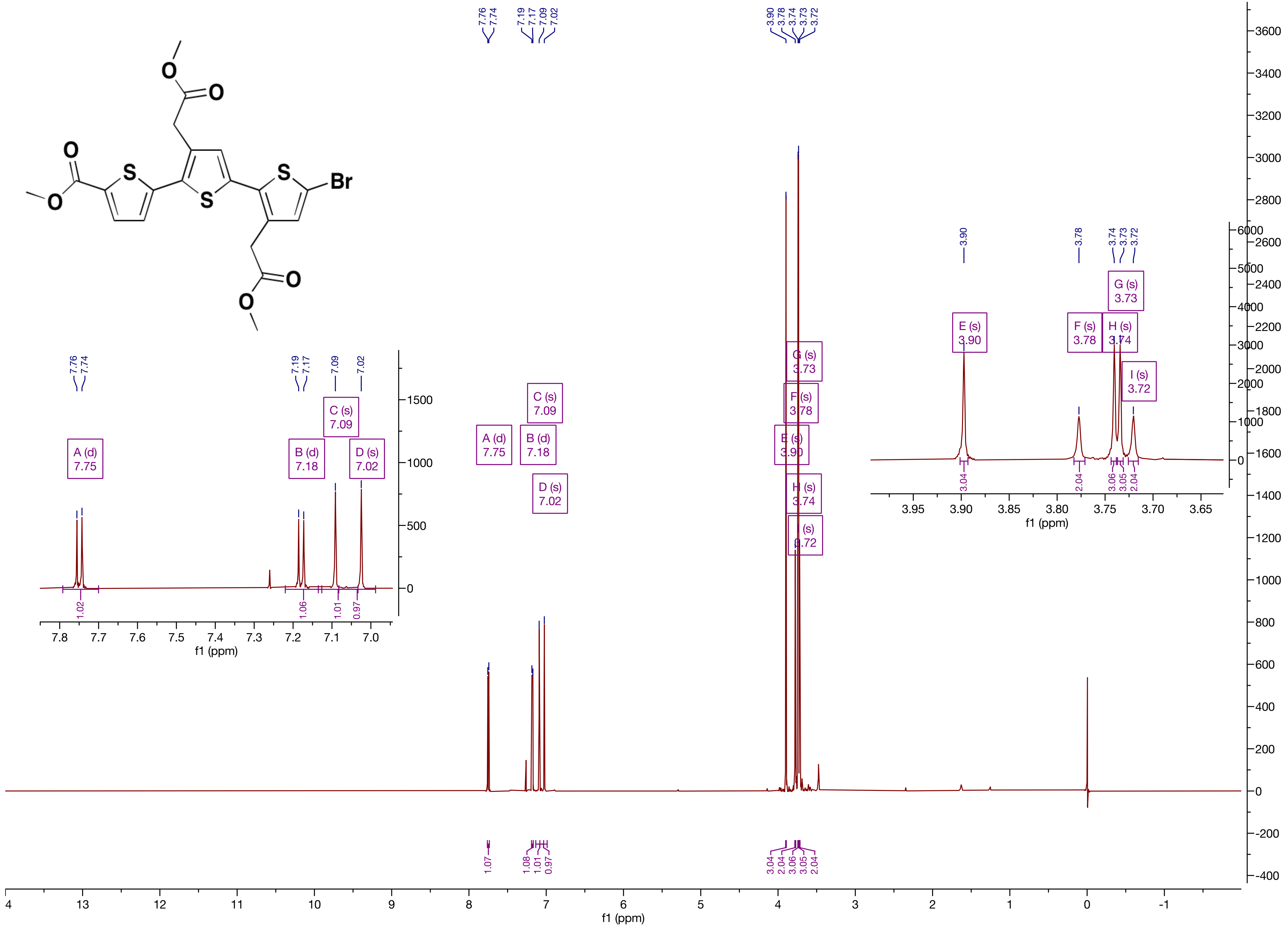

O
O
O
S
S
S
Br
O
O
O
7.76
7.74
7.19
7.17
7.09
7.02
3.90
3.78
3.74
3.73
3.72
A (d) 7.75
B (d) 7.18
C (s) 7.09
D (s) 7.02
E (s) 3.90
F (s) 3.78
G (s) 3.73
H (s) 3.74
I (s) 3.72
1.02
1.06
1.01
0.97
3.04
2.04
3.06
3.05
2.04
1.07
1.08
f1 (ppm)

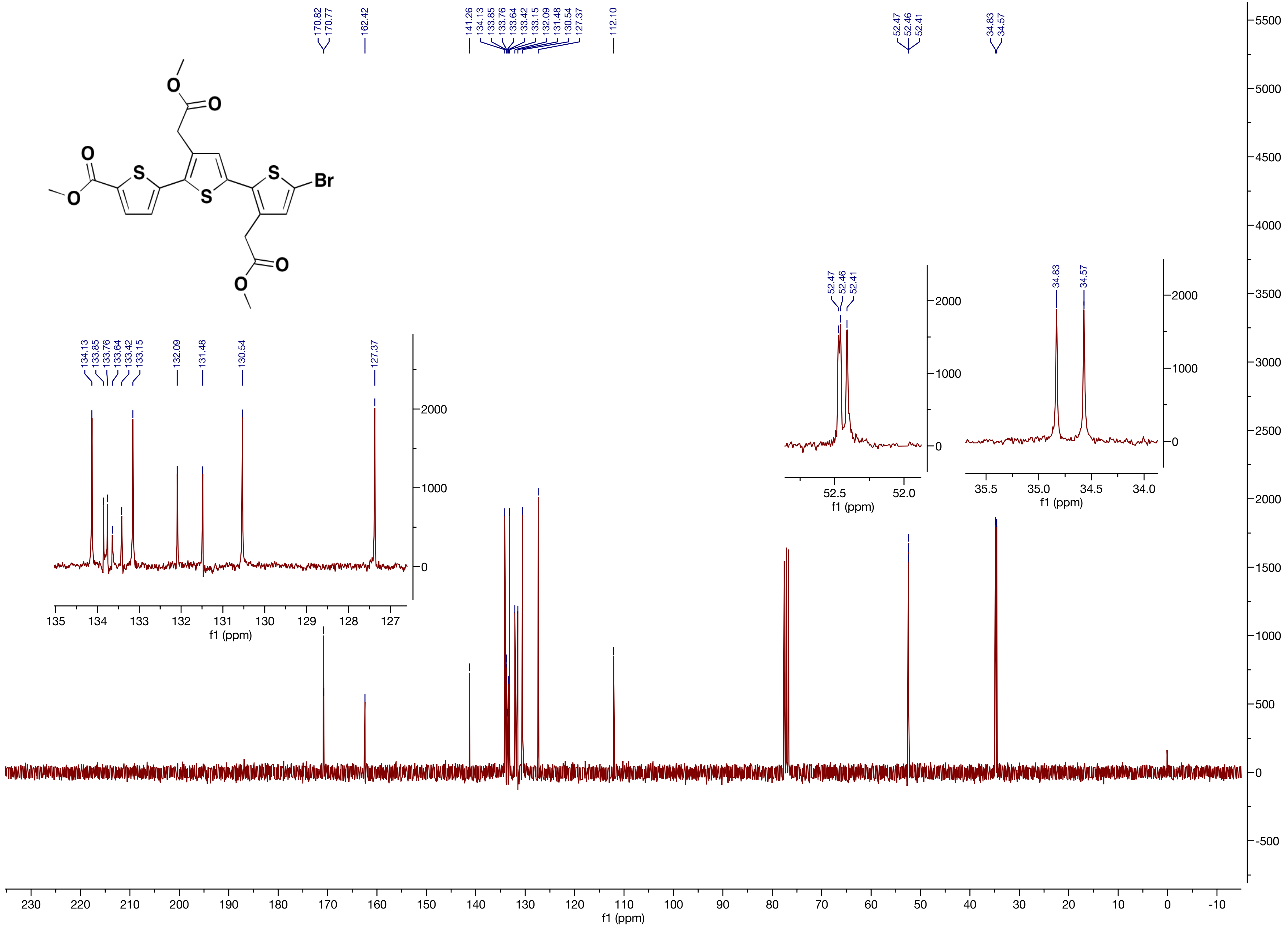

170.82
170.77
162.42
141.26
134.13
133.85
133.76
133.64
133.42
133.15
132.09
131.48
130.54
127.37
112.10
52.47
52.46
52.41
34.83
34.57
S
S
S
Br
O
f1 (ppm)

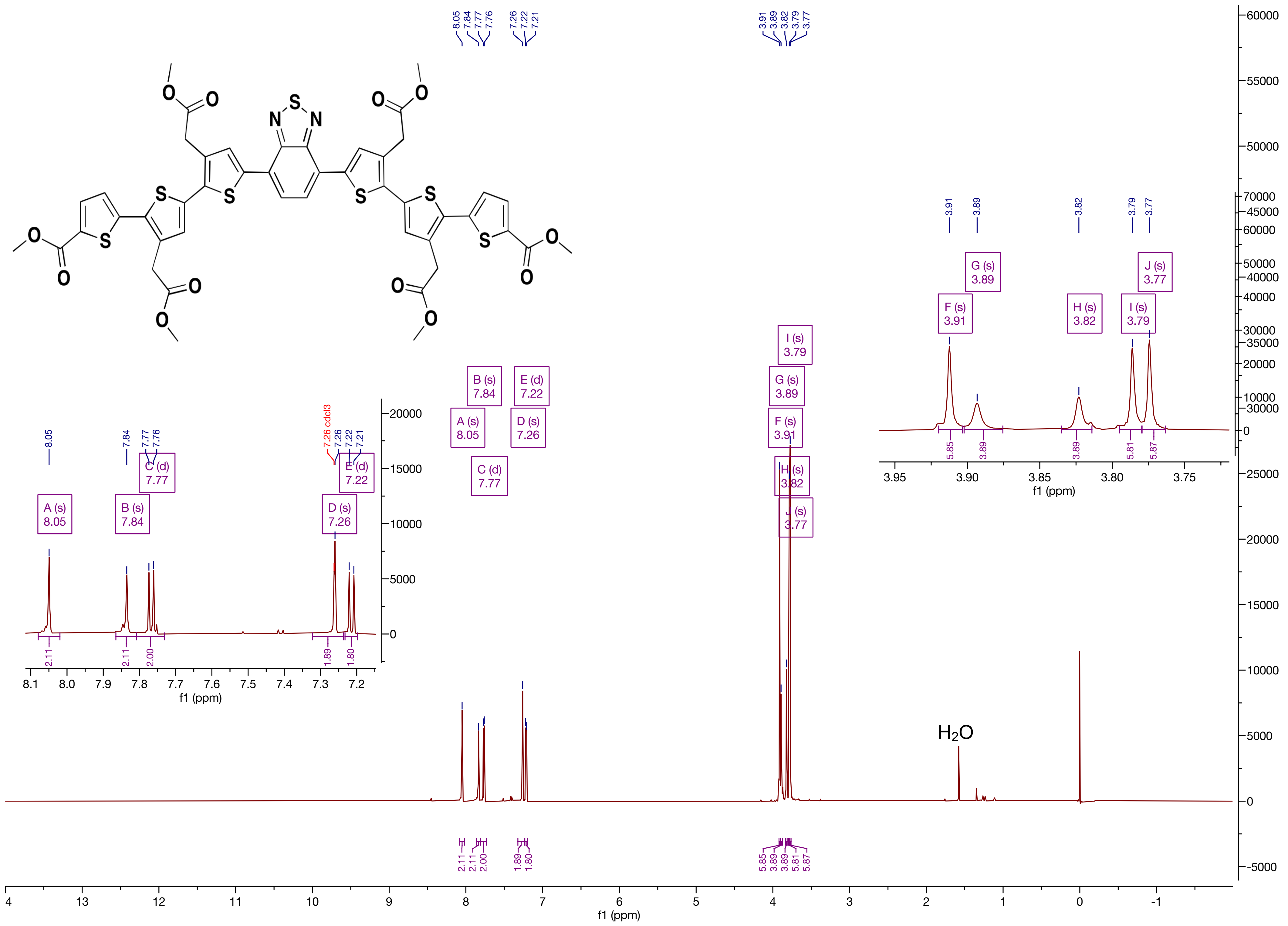

8.05 7.84 7.77 7.76 7.26 7.22 7.21
3.91 3.89 3.82 3.79 3.77
A (s) 8.05
B (s) 7.84
C (d) 7.77
D (s) 7.26
E (d) 7.22
F (s) 3.91
G (s) 3.89
H (s) 3.82
I (s) 3.79
J (s) 3.77
7.26 cdcl3
2.11 2.11 2.00 1.89 1.80
5.85 3.89 3.89 5.81 5.87
H2O
f1 (ppm)

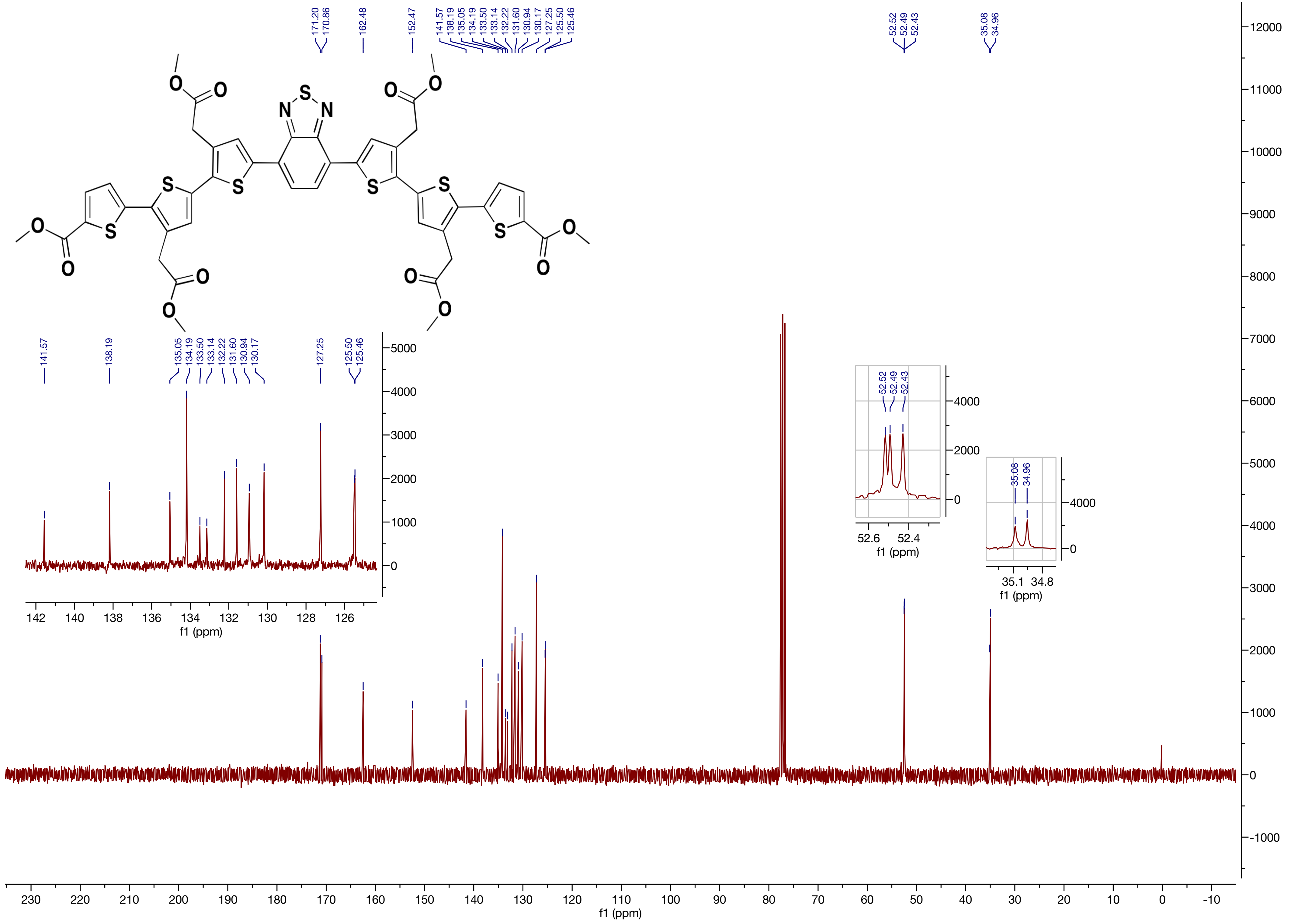

171.20
170.86
162.48
152.47
141.57
138.19
135.05
134.19
133.50
133.14
132.22
131.60
130.94
130.17
127.25
125.50
125.46
52.52
52.49
52.43
35.08
34.96
f1 (ppm)
